# Efficient Partitioning of Memory Systems and Its Importance for Memory Consolidation

**DOI:** 10.1371/journal.pcbi.1003146

**Published:** 2013-07-25

**Authors:** Alex Roxin, Stefano Fusi

**Affiliations:** 1 Center for Theoretical Neuroscience, Columbia University, New York, New York, United States of America; 2 Centre de Recerca Matemàtica, Campus de Bellaterra, Bellaterra, Barcelona, Spain; Duke University, United States of America

## Abstract

Long-term memories are likely stored in the synaptic weights of neuronal networks in the brain. The storage capacity of such networks depends on the degree of plasticity of their synapses. Highly plastic synapses allow for strong memories, but these are quickly overwritten. On the other hand, less labile synapses result in long-lasting but weak memories. Here we show that the trade-off between memory strength and memory lifetime can be overcome by partitioning the memory system into multiple regions characterized by different levels of synaptic plasticity and transferring memory information from the more to less plastic region. The improvement in memory lifetime is proportional to the number of memory regions, and the initial memory strength can be orders of magnitude larger than in a non-partitioned memory system. This model provides a fundamental computational reason for memory consolidation processes at the systems level.

## Author Summary

Memory is critical to virtually all aspects of behavior, which may explain why memory is such a complex phenomenon involving numerous interacting mechanisms that operate across multiple brain regions. Many of these mechanisms cooperate to transform initially fragile memories into more permanent ones (memory consolidation). The process of memory consolidation starts at the level of individual synaptic connections, but it ultimately involves circuit reorganization in multiple brain regions. We show that there is a computational advantage in partitioning memory systems into subsystems that operate on different timescales. Individual subsystems cannot both store large amounts of information about new memories, and, at the same time, preserve older memories for long periods of time. Subsystems with highly plastic synapses (fast subsystems) are good at storing new memories but bad at retaining old ones, whereas subsystems with less plastic synapses (slow subsystems) can preserve old memories but cannot store detailed new memories. Here we propose a model of a multi-stage memory system that exhibits the good features of both its fast and its slow subsystems. Our model incorporates some of the important design principles of any memory system and allows us to interpret in a new way what we know about brain memory.

## Introduction

Memories are stored and retained through a series of complex, highly coupled processes that operate on different timescales. In particular, it is widely believed that after the initial encoding of a sensory-motor experience, a series of molecular, cellular, and system-level alterations lead to the stabilization of an initial memory representation (memory consolidation). Some of these alterations occur at the level of local synapses, while others involve the reorganization and consolidation of different types of memories in different brain areas. Studies of patient HM revealed that medial temporal lobe lesions severely impair the ability to consolidate new memories, whereas temporally remote memories remain intact [1]. These results and more recent work (see e.g. [2]) suggest that there may be distinct memory systems, and that memories, or some of their components, are temporarily stored in the medial temporal lobe and then transferred to other areas of the cortex. Is there any fundamental computational reason for transferring memories from one area to another? Here we consider memory models consisting of several stages, with each stage representing a region of cortex characterized by a particular level of synaptic plasticity. Memories are continuously transferred from regions with more labile synapses to regions with reduced but longer-lasting synaptic modifications. Here we refer to each region as a stage in the memory transfer process. We find that such a multi-stage memory model significantly outperforms single-stage models, both in terms of the memory lifetimes and the strength of the stored memory. In particular, memory lifetimes are extended by a factor that is proportional to the number of memory stages.

In a memory system that is continually receiving and storing new information, synaptic strengths representing old memories must be protected from being overwritten during the storage of new information. Failure to provide such protection results in memory lifetimes that are catastrophically low, scaling only logarithmically with the number of synapses [3]–[5]. On the other hand, protecting old memories too rigidly causes memory traces of new information to be extremely weak, being represented by a small number of synapses. This is one of the aspects of the classic plasticity-rigidity dilemma (see also [6]–[8]). Synapses that are highly plastic are good at storing new memories but poor at retaining old ones. Less plastic synapses are good at preserving memories, but poor at storing new ones.

A possible solution to this dilemma is to introduce complexity into synaptic modification in the form of metaplasticity, by which the degree of plasticity at a single synapse changes depending on the history of previous synaptic modifications. Such complex synapses are endowed with mechanisms operating on many timescales, leading to a power-law decay of the memory traces, as is widely observed in experiments on forgetting [9], [10]. Furthermore, complex synapses can vastly outperform previous models due to an efficient interaction between these mechanisms [11]. We now show that allowing for a diversity of timescales can also greatly enhance memory performance at the systems level, even if individual synapses themselves are not complex. We do this by considering memory systems that are partitioned into different regions, the stages mentioned above, characterized by different degrees of synaptic plasticity. In other words, we extend the previous idea of considering multiple timescales at single synapses to multiple timescales of plasticity across different cortical areas.

To determine how best to partition such a memory system, we take the point of view of an engineer who is given a large population of synapses, each characterized by a specific degree of plasticity. Because we want to focus on mechanisms of memory consolidation at the systems level, we use a simple binary model in which synaptic efficacies take two possible values, weak and strong. Previous work has shown that binary synapses are representative of a much wider class of more realistic synaptic models [5]. It seems likely that the mechanisms for storing new memories exploit structural aspects and similarities with previously stored information (see e.g. semantic memories). In our work, we are interested in different mechanisms responsible for storing new information that has already been preprocessed in this way and is thus incompressible. For this reason, we restrict consideration to memories that are unstructured (random) and do not have any correlation with previously stored information (uncorrelated). After constructing multi-state models, we estimate and compare their memory performance both in terms of memory lifetime and the overall strength of their memory traces.

## Results

### The importance of synaptic heterogeneity

**Figure 1:**
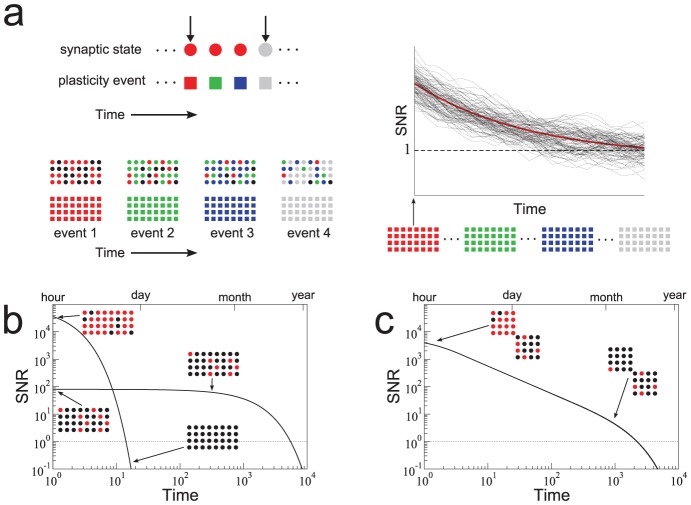
Heterogeneity in synaptic learning rates is desirable. **a.** Upper left: Each synapse is updated stochastically in response to a plasticity event, and encodes one bit of information of one specific memory because it has only two states. For this reason, we can assign a color to each synapse which represents the memory that is stored. Lower left: Memories are encoded by subjecting 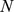 synapses to a pattern of plasticity events, here illustrated by different colors. These patterns, and hence the memories, are random and uncorrelated. The strength of a memory is defined as the correlation between the pattern of synaptic weights and the event being tracked. The degradation of encoded memories is due to the learning of new memories. Only four memories are explicitly tracked in this example: red, green, blue, gray. Those synapses whose state is correlated with previous memories are colored black. Right: Synaptic updating is stochastic in the model leading to variability in the signal for different realizations given the same sequence of events (dotted lines). A mean-field description of the stochastic dynamics captures signal strength averaged over many realizations. We measure the signal-to-noise ratio (SNR) which is the signal relative to fluctuations in the overlap of uncorrelated memories. **b.** There is a trade-off between the initial SNR and the memory lifetime: A large initial SNR can be achieved if the probability 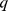 of a synapse changing state is high (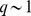), although the decay is rapid, i.e. the memory lifetime scales as 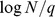, where 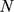 is the total number of synapses. Long lifetimes can be achieved for small 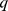 although the initial SNR is weak. Memory lifetime can be as large as 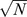, when 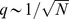. SNR vs time curves are shown for 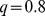 and 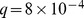 and 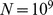. **c.** In a heterogeneous population of synapses in which many *q*s are present, one can partially overcome the trade-off (black line). The initial SNR scales as 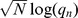, where 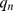 is the learning rate corresponding to the slowest population. The memory lifetime scales as 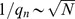. Here there are 50 different *q*s, 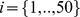 where 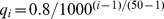 and 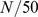 synapses of each type. 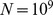.

We first analyzed a homogeneous model (single partition), in which all the synapses have the same learning rate (see Fig. 1). We consider a situation in which new uncorrelated memories are stored at a constant rate. Synapses are assumed to be stable in the absence of any overwriting due to the learning of new memories. Each memory is stored by modifying a randomly selected subset of synapses. As the synapses are assumed to be bistable, we reduce all the complex processes leading to long term modifications to the probability that a synapse makes a transition to a different state. As memories are random and uncorrelated, the synaptic transitions induced by different memories will be stochastic and independent.

To track a particular memory we take the point of view of an ideal observer who has access to the values of the strengths of all the synapses relevant to a particular memory trace (see also [11]). Of course in the brain the readout is implemented by complex neural circuitry, and the estimates of the strength of the memory trace based on the ideal observer approach provide us with an upper bound of the memory performance. However, given the remarkable memory capacity of biological systems, it is not unreasonable to assume that specialized circuits exist which can perform a nearly optimal readout, and we will describe later a neural circuit that replicates the performance of an ideal observer.

More quantitatively, to track a memory, we observe the state of an ensemble of 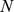 synapses and calculate the memory signal, defined as the correlation between the state of the ensemble at a time 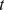 and the pattern of synaptic modifications induced by the event of interest at time 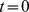. Specifically, we can formalize this model description by assigning the value 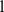 to a potentiated synapse and 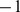 to a depressed one. Similarly, a plasticity event is assigned a value 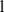 if it is potentiating and 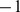 if depressing. We then define a vector of length 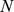, 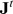 where 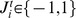 is the state of synapse 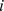 at time 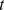. Similarly, the memories are also vectors of length 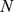, 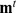, where 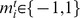 is the plasticity event to which synapse 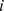 is subjected at time 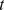. If we choose to track the memory presented at time 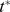, then we define the memory trace as the signal at time 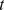, which is just the dot product of two vectors, 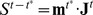. The signal itself is a stochastic variable, since the updating of the synaptic states is stochastic. This means that if one runs several simulations presenting exactly the same memories, the signal will be different each time, see right hand side of Fig. 1a. The mean signal, understood as the signal averaged over many realizations of the Markov process, can be computed analytically. For the homogeneous model, a continuous-time approximation to the mean signal takes the simple form of an exponential, 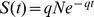, where 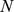 is the total number of synapses and 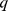 is the learning rate, see *Methods* and *Text S1* for details. We must compare this mean signal to the size of fluctuations in the model, i.e the noise.

The memory noise is given by the size of fluctuations in the overlap between uncorrelated patterns, which here is approximately 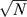, see *Text S1* for details. Therefore, the signal-to-noise ratio 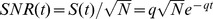. One can track a particular memory only until it has grown so weak it cannot be discerned from any other random memory. Memory lifetime, which is one measure of the memory performance, is then simply defined as the maximum time over which a memory can be detected. More quantitatively it is the maximum time over which the SNR is larger than 1. The scaling properties of the memory performance that we will derive do not depend on the specific critical SNR value that is chosen. Moreover, it is known that the scaling properties derived from the SNR are conserved in more realistic models of memory storage and memory retrieval with integrate-and-fire neurons and spike driven synaptic dynamics (see e.g. [12]).

As we mentioned, the dynamics of the Markov model we consider are stochastic. Therefore, throughout the paper, we will discuss results from stochastic models for which we have derived corresponding mean-field descriptions. Fig. 1b shows the mean-field result for two extreme cases when all synapses have the same degree of plasticity. If the synapses are fast and the transition probability is high (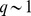), then the memory is very vivid immediately after it is stored and the amount of information stored per memory is large, as indicated by the large initial SNR (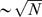). However the memory is quickly overwritten as new memories are stored. In particular, the memory lifetime scales as 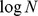 which is extremely inefficient: doubling the lifetime requires squaring the number of synapses.

It is possible to extend lifetimes by reducing the learning rate, and in particular by letting the learning rate scale with the number of synapses. For the smallest 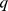 that still allows one to store sufficient information per memory (i.e. that allows for an initial SNR∼1), 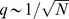, the memory lifetimes are extended by a factor that is proportional to 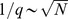. This trade-off between memory lifetime and initial SNR (i.e. the amount of information stored per memory) cannot be circumvented through the addition of a large number of synaptic states without fine-tuning the balance of potentiation and depression [5].

These shortcomings can be partially overcome by allowing for heterogeneity in the transition probabilities within an ensemble of synapses. Specifically, if there are 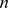 equally sized groups of synapses, each with a different transition probability 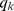 (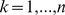), then the most plastic ones will provide a strong initial SNR while the least plastic ones will ensure long lifetimes. Intermediate time-scales are needed to bridge the gap between the extreme values. In Fig. 1c we plot the SNR as a function of time. Transition probabilities are taken to be of the form 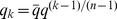, where 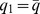 is the fastest learning rate, 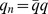 is the slowest learning rate and 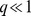. Time is expressed in terms of the number of uncorrelated memories on the lower axis, and we choose an arbitrary rate of new uncorrelated memories (one per hour) to give an idea of the different orders of magnitudes of the timescales that are at play (from hours to years). This model, which we call *the heterogeneous model* is already an interesting compromise in terms of memory performance: as we increase the number of synapses, if the slowest learning rate is scaled as 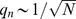, then both the initial SNR and the memory lifetime scale advantageously with the number of synapses (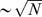). Moreover, the model has the desirable property that the memory decay is a power law over a wide range of timescales, as observed in several experiments on forgetting [13].

### The importance of memory transfer

In the heterogeneous model, the synapses operate on different timescales independently from each other. We now show that the performance can be significantly improved by introducing a feed-forward structure of interactions from the most plastic group to the least plastic group of synapses. How is this possible? While the least plastic synapses can retain memories for long times, their memory trace is weak. However, this memory trace can be boosted through periodic rewriting of already-stored memories. If a memory is still present in one of the groups of synapses (called hereafter a ‘memory stage’), the stored information can be used to rewrite the memory in the downstream stages, even long after the occurrence of the event that created the memory.

It is important to notice that not all types of rewriting can significantly improve all the aspects of the memory performance. For example, if all memories are simply reactivated the same number of times, then the overall learning rate changes, so that the initial memory trace becomes stronger, but the memory lifetimes are reduced by the same factor. Rather, an alternative strategy is to reactivate and rewrite a combination of multiple memories, one which has a stronger correlation with recent memories and a weaker correlation with the remote ones.

**Figure 2:**
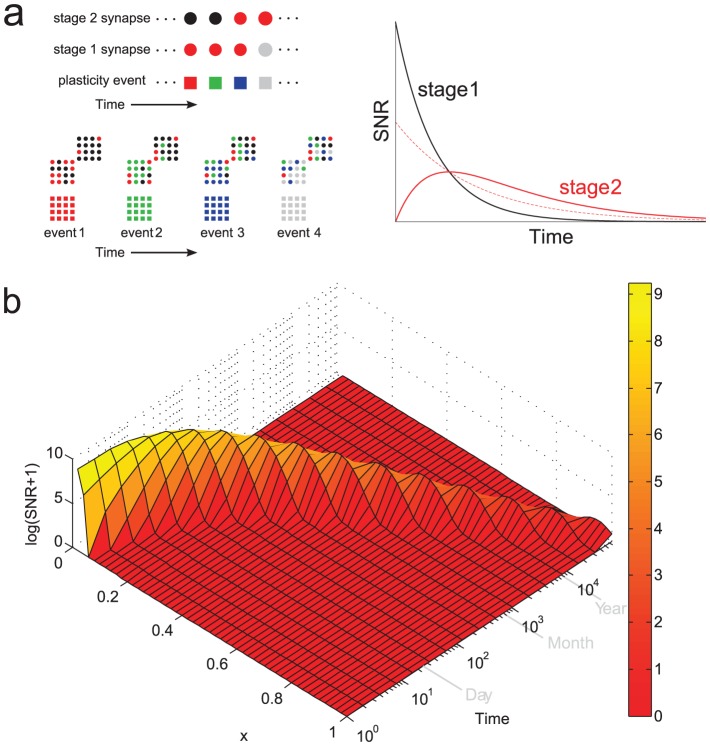
The memory transfer model. **a.** Upper left: In the model, the state of each synapse in stage one is updated stochastically in response to the occurrence of plasticity events. The synapses of downstream stages update their state according to the state of upstream stages. Lower left: Memories are encoded by subjecting the 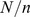 synapses in stage 1 of 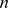 stages to a pattern of plasticity events, here illustrated by different colors. The correlation of synaptic states with a memory is initially zero in downstream stages, and builds up over time through feed-forward interactions. Right: The consolidation model always outperforms the heterogeneous model without interactions at sufficiently long times. Here a two-stage model is illustrated. The dashed line is the SNR of the second stage in the heterogeneous model. See text for details. **b.** The memory wave: the memory trace (from Eq. 1) in the consolidation model travels as a localized pulse from stage to stage (starting from 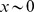, in fast learning stages, presumably localized in the medial temporal lobe, and ending at 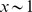, in slow learning stages). Here 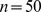 and 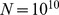. Stage 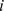 has a learning rate 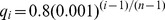 and 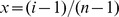. New memories are encoded at a rate of one per hour.

We have built a model, which we will call *the memory transfer model*, that implements this idea. We consider 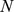 synapses divided into 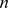 interacting stages. We assume that all the stages have the same size and that synapse 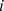 in stage 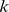 can influence a counterpart synapse 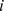 in stage 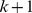. In particular, synapses in the first stage undergo stochastic event-driven transitions as before (Fig. 2a). They therefore encode each new memory as it is presented. On the other hand, synapses in downstream stages update their state stochastically after each memory is encoded in the first stage.

Specifically, at time 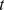, a memory 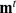 of length 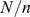 consisting of a random pattern of potentiating (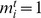) and depressing (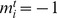) events is presented to the 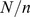 synapses in stage one, which have synaptic state 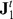. Synapse 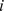 is subjected either to a potentiating (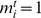) or to a depressing (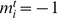) event with probability 1/2, and is updated with a probability 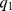 as in the previous models. Therefore, the updating for synapses in stage 1 is identical to that for ensemble 1 in the synaptic model with heterogeneous transition probabilities which we discussed previously. Now, however, we assume that a synapse 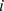 in stage 2 is influenced by the state of synapse 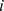 in stage 1 in the following way. If synapse 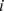 in stage 1 is in a potentiated (depressed) state at time 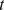 (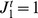 or 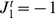 respectively), then synapse 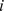 in stage 2 will potentiate (depress) at time 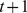 with probability 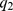. The update rule for synapses in stage 3 proceeds analogously, but depends now on the state of synapses in stage 2, and so on.

In other words, after each memory is stored, a random portion of the synaptic matrix of each stage is copied to the downstream stages with a probability that progressively decreases. We will show later that this process of “synaptic copying” can actually be mediated by neuronal activity which resembles the observed replay activity [14]–[19]. Transition probabilities of the different memory stages are the same as in the heterogeneous model: 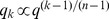. We will follow the SNR for a particular memory by measuring the correlation of the synaptic states in each stage with the event of interest.

Once again, we can derive a mean-field description of the stochastic dynamics. The upshot is that the mean signal in stage 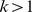 obeys the differential equation
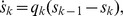
which expresses clearly how the signal in stage 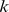 is driven by that in stage 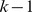. This is precisely the mechanism behind the improvement of memory performance compared to the heterogenous model without interactions. The memory trace in the first stage decays exponentially as new memories are encoded, as in the homogeneous case (see Fig. 2a). Memory traces in downstream stages start from zero, increase as the synaptic states are propagated, and eventually decay once again to zero. Information about all the stored memories is transferred between stages because the synapses that are “copied” are correlated to all the memories that are still represented at the particular memory stage. The most plastic stages retain the memories for a limited time, but during this time they transfer them to less plastic stages. This explains why the memory traces of downstream stages are non-monotonic functions of time: at stage 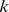, the memory trace keeps increasing as long as the information about the tracked memory is still retained in stage 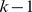. The memory trace in the second stage is already greater than that of an equivalent heterogeneous model with independent synaptic groups (Fig. 2a). This effect is enhanced as more stages are added.

The memory trace takes the form of a localized pulse that propagates at an exponentially decreasing rate (Fig. 2b). It begins as a sharply peaked function in the fast learning stages but slowly spreads outward as it propagates toward the slow learning stages. This indicates that although the memory is initially encoded only in the first stage (presumably located in the medial temporal lobe), at later times it is distributed across multiple stages. Nonetheless, it has a well defined peak, meaning that at intermediate times the memory is most strongly represented in the synaptic structure of intermediate networks.

**Figure 3:**
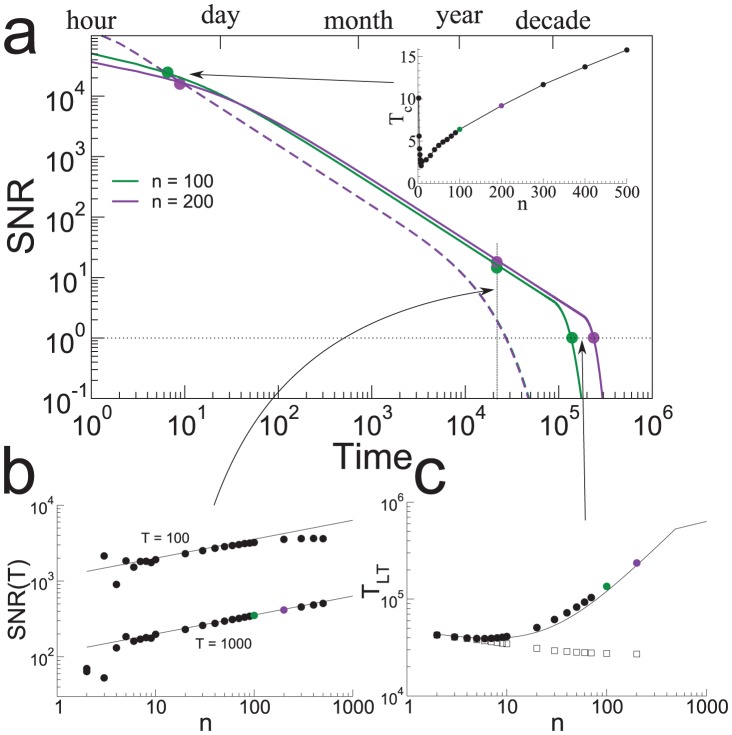
The consolidation model yields long lifetimes and large SNR. **a.** The SNR for two values of 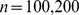 for a fixed number of synapses (solid lines: consolidation model, dotted lines: heterogeneous model without interactions). The initial SNR for both models scales as 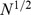. It then decays as power law (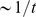) and finally as an exponential for 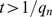 for the heterogeneous model and for 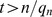 for the consolidation model. Three measures of interest are shown in the inset and in the bottom two panels. Inset: crossing time 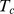 between the SNR of the heterogeneous model and the SNR of the consolidation model as a function of 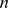. The heterogeneous model is better than the consolidation model only for very recent memories (stored in the last hours, compared to memory lifetimes of years). **b.** The SNR scales as 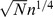 in the consolidation model when the SNR decay is approximately a power law (symbols: simulations, line: analytics). The SNR at indicated times is plotted as a function of 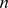 for three different values of 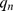. **c.** Lifetimes (i.e. time at which SNR = 1) in the consolidation model scale approximately as 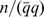 (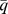 is the fastest learning rate and 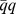 is the slowest). The memory lifetime is plotted vs 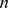 for three different values of 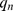. 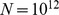 synapses evenly divided into 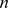 stages. Stage 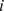 has a learning rate 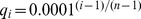.

An analytical formula for the pulse can be derived, see *Methods* and *Text S1*, which allows us to calculate the SNR and memory lifetimes (Fig. 3). Now, when reading out the signal from several stages of the memory transfer model, we must take into account the fact that the noise will be correlated. This was not the case for the heterogeneous model without interactions. In fact, if we consider a naive readout which includes all 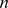 stages, the noise will increase weakly with the number of stages. On the other hand, if we only read out the combination of stages which maximizes the SNR, one can show that the noise is independent of 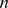 and very close to the uncorrelated case. In fact, this readout is equivalent to reading out only those groups whose SNR exceeds a fixed threshold, which could be learned, see *Text S1* for more details.

Fig. 3a shows the SNR for memories in the heterogeneous model (dashed lines) and the memory transfer model (solid lines) for a fixed number of synapses and different numbers of groups 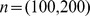. The curves are computed using the optimal readout described above, for which noise correlations are negligible. Both the SNR for intermediate times and the overall lifetime of memories increase with increasing 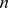 in the memory transfer model. The increase in SNR is proportional to 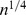, see Fig. 3b, while the lifetime is approximately linear in 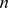 for large enough 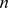, see Fig. 3c. While the initial SNR is reduced compared to the heterogeneous model (by a factor proportional to 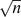), it overtakes the SNR of the heterogeneous model already at very short times (inset of Fig. 3a).

Importantly, the memory transfer model also maintains the propitious scaling seen in the heterogeneous model of the SNR and memory lifetime with the number of synapses 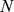. Specifically, if the slowest learning rate is scaled as 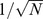, then the very initial SNR scales as 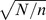 (but almost immediately after the memory storage it scales as 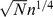) and the lifetime as 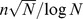. Hence the lifetime is extended by a factor that is approximately 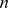 with respect to the memory lifetime of both the heterogeneous model and the cascade synaptic model [11] in which the memory consolidation process occurs entirely at the level of individual complex synapses. The improvement looks modest on a logarithmic scale, as in Fig. 3a, however it becomes clear that it is a significant amelioration when the actual timescales are considered. In the example of Fig. 3a the memory lifetime extends from three years for the heterogeneous model, to more than thirty years for the memory transfer model. As the memory lifetime extends, the initial signal to noise ratio decreases compared to the heterogeneous model (but not compared to the cascade model, for which it decreases as 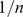, where 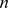 is the number of levels of the cascade, or in other words, the complexity of the synapse). However, the 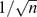 reduction is small, and after a few memories the memory transfer model already outperforms the heterogeneous model. In the example of Fig. 3 the heterogeneous model has a larger SNR only for times of the order of hours. This time interval should be compared to the memory lifetime which is of the order of decades.

### Neuronal implementation and the role of replay activity

**Figure 4:**
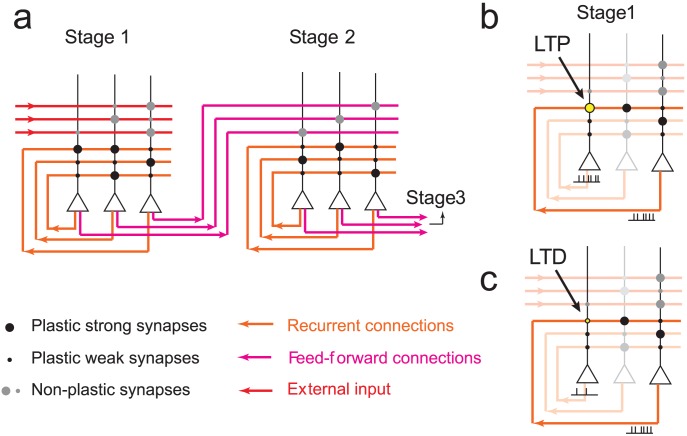
The neural network implementing the memory transfer model. **a** A schematic representation of the neural network architecture. Here we show stage 1 and 2, but the other memory stages are wired in the same way. Neurons are represented by triangles and synaptic connections by circles. The axons are red, purple and orange, and the dendritic trees are black. Each neuron connects to all the neurons in the same stage (recurrent connections, orange) and to the corresponding neuron in the downstream stage (feed-forward connections, purple). The recurrent connections are plastic whereas the feed-forward connections are fixed. **b,c** memory encoding: a pattern of activity is imposed in the first stage only and synapses are updated stochastically according to a Hebbian rule. Specifically, if both the pre and the post-synaptic neurons are simultaneously active (b, the activity is schematically represented by trains of spikes), the synapse is potentiated. If the pre-synaptic neuron is active and the post-synaptic neuron is inactive, the synapse is depressed (c).

The consolidation model we have described involves effective interactions between synapses that must be mediated by neuronal activity. We now show that it is possible to build a neuronal model that implements these interactions. We consider a model of 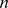 identical stages, each one consisting of 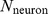 recurrently connected McCulloch-Pitts neurons (the total number of plastic synapses is 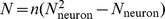). Neurons in each stage are connected by non-plastic synapses to the corresponding neurons in the next stage (feed-forward connections). See Fig. 4a for a scheme of the network architecture. The model operates in two different modes: encoding and transfer. Importantly, we must now be more careful concerning our definition of time. The unit of time we have used up until now was simply that of the encoding of a memory, i.e. one time step equals one memory. Now we have two different time scales: the encoding time scale and the neuronal time scale. The encoding time scale is just the same as before, i.e. it is the time between learning new memories. The neuronal time scale is much faster. Specifically, in the neuronal model we encode a new memory and then stimulate the neurons to drive the transfer of patterns of synaptic weights. The time-step used in the Hebbian learning process when a memory is encoded, as well as the time-step used during this transfer process is a neuronal time scale, perhaps from milliseconds to hundreds of milliseconds. The time between memory encodings, on the other hand, might be on the order of minutes or hours, for example.

During encoding, a subset of neurons in the first stage is activated by the event that creates the memory and the recurrent synapses are updated according to a Hebbian rule, see Fig. 4b,c. Specifically, one half of the neurons are randomly chosen to be activated (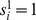), while the remaining neurons are inactive (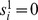), where 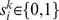 is the state of the neuron 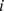 in stage 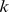. A synapse 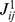 is then potentiated (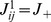) with a probability 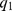 if 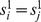 and is depressed (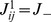) with probability 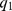 if 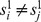, where 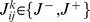 is a binary synapse from neuron 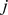 to neuron 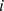 in stage 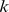. Consistent with the previous analysis, we assume that the neuronal patterns of activity representing the memories are random and uncorrelated. No plasticity occurs in the synapses of neurons in downstream stages during encoding.

During transfer, a random fraction 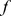 of neurons in each stage is activated at one time step, and the network response then occurs on the following time-step due to recurrent excitatory inputs. Specifically, at time 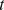, 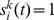 for all 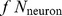 neurons which have been activated in stage 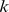, and otherwise 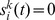. At time 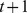 the recurrent input to a neuron 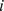 in stage 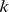 due to this activation is 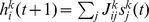. If 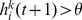 then 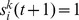 and otherwise 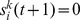, where 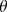 is a threshold. At time 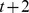 all neurons are silenced, i.e. 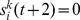 and then the process is repeated 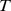 times. The initially activated neurons at time 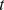 are completely random and in general they will not be correlated with the neuronal representations of the stored memories. However, the neuronal response at time 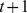 will be greatly affected by the recurrent synaptic connections. For this reason, the activity during the response will be partially correlated with the memories stored in the upstream stages, similar to what happens in observed replay activity (see e.g. [14]–[19]).

During transfer, the activated neurons project to counterpart neurons in the downstream stage. Crucially, we assume here that the long-range connections from the upstream stage to the downstream one are up-regulated relative to the recurrent connections in the downstream stage. In this way, the downstream state is “taught” by the upstream one. In the brain this may occur due to various mechanisms which include neuromodulatory effects and other gating mechanisms that modulate the effective couplings between brain regions. Cholinergic tone, in particular, has been shown to selectively modulate hippocampal and some recurrent cortical synapses (see [20]) as well as thalamocortical synapses [21]. Recent studies have also shown that the interactions between cortical and subcortical networks could be regulated by changing the degree of synchronization between the rhythmic activity of different brain areas (see e.g. [22]).

In our model we assumed that, due to strong feedforward connections, whenever 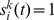 we have 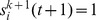. The pattern of activation in stage 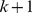 therefore follows that of stage 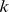 during the transfer process. Importantly plasticity only occurs in the recurrent synapses of the *downstream* stage 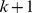, i.e. stage 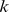 is ‘teaching’ stage 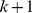. For illustration we first consider a simple learning rule which can perfectly copy synapses from stage 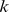 to stage 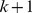, but only for the special case of 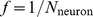, i.e. single-neuron stimulation. Following this, we will consider a learning rule which provides for accurate but not perfect copying of synapses but which is valid for any 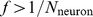.

**Figure 5:**
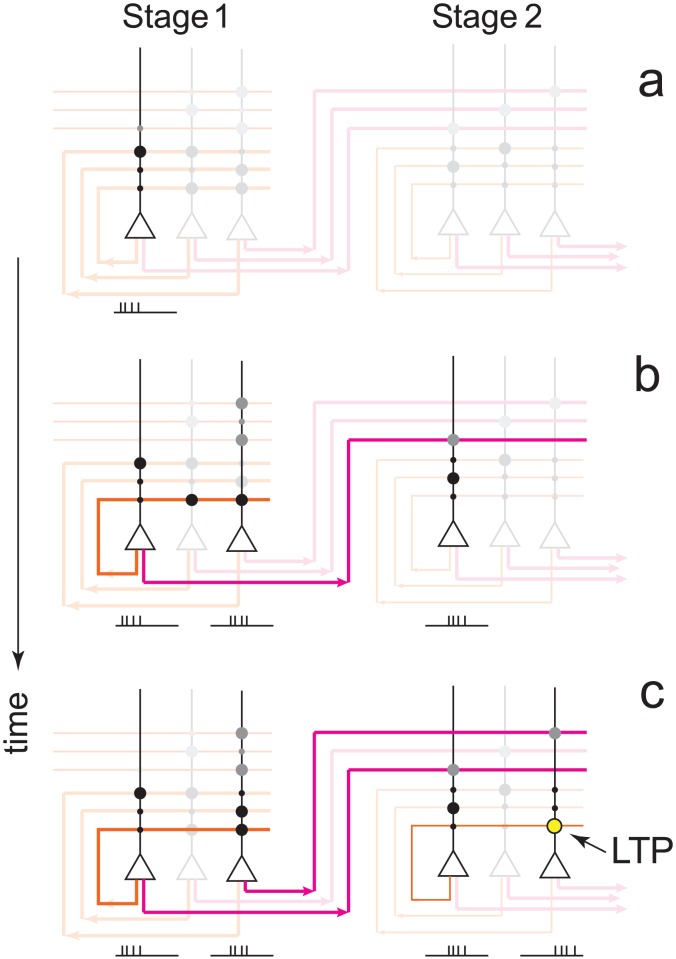
A schematic example of the transfer process. Synapses are here transferred from stage 1 to stage 2, the same mechanism applies to any other two consecutive stages. During the transfer process the feed-forward connections are up-regulated, and the recurrent connection of the target stage are down-regulated. The process starts with the stimulation of a fraction 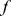 of randomly chosen neurons in stage 1 (a). The activity of the neuron is schematically represented by a train of spikes. The axon branches of the activated neurons (in this example only one neuron is activated) are highlighted. (b) the spontaneous activation of neuron 1, causes the activation of the corresponding neuron in stage 2 and of the stage 1 neurons that are most strongly connected. The process of relaxation has started. (c) the recurrently connected neurons of stage 1 which are activated, excite and activate the corresponding neurons in stage 2. As a result of the consecutive activation of the two highlighted neurons in stage 2, the synapse pointed by an arrow is potentiated, ending up in the same state as the corresponding synapse in stage 1. The strength of one synapse in stage 1 has been successfully copied to the corresponding synapse in stage 2.

Fig. 5 shows a schematic of the transfer process when 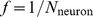. In this simplest case, only one presynaptic synapse per neuron is activated. To successfully transfer this synapse to the downstream stage a simple rule can be applied. First, the threshold is set so that 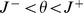. If there is a presynaptic spike (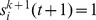) followed by a postsynaptic spike (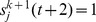), then potentiate (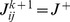) with a probability equal to the intrinsic learning rate of the synapses, 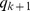. If there is no postsynaptic spike (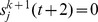) then the corresponding synapse should be depressed (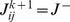). This leads to perfect transfer.

In general 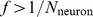 and therefore it is not possible to perfectly separate inputs with a single threshold. Nevertheless, a learning rule which can accurately copy the synapses in this general case is the following. Consider two thresholds 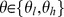, which are ‘low’ and ‘high’ respectively. On any given transfer (there are 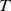 of them per stage) 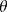 is set to one of these two thresholds with probability 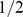. If 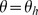 then if 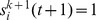 and 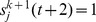, then set 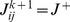 with a probability 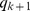. In words, this says that if despite the high threshold, the presynaptic activity succeeded in eliciting postsynaptic activity, then the synapses in stage 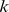 must have been strong, therefore one should potentiate the corresponding synapses in stage 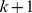. Similarly if 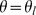 then if 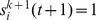 and 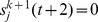, then set 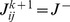 with a probability 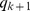. In words, this says that if despite the low threshold, the presynaptic activity did not succeed in eliciting postsynaptic activity, then the synapses in stage 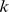 must have been weak, therefore one should depress the corresponding synapses in stage 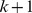. For this learning rule to work, both stages 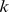 and 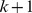 must be privy to the value of the threshold. Therefore, there must be some global (at least common to these two stages) signal available. This could be achieved via a dynamical brain state with long-range spatial correlations. For example, globally synchronous up-state and down-state transitions [23], which are known to occur during so-called slow-wave sleep would be ideally suited to shift neuronal thresholds. Alternatively, theta oscillations have been shown to be coherent between hippocampus and prefrontal cortex in awake behaving rodents during working memory [24] and learning tasks [25] and would also be suited to serve as a global signal for synaptic plasticity.

We have stated that this second learning rule involving two thresholds can lead to accurate learning in the general case. Concretely, we can completely characterize the transfer process between any two stages via two quantities: the transfer rate 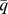, which is the fraction of synapses transferred after 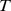 replays of the transfer process, and the accuracy of transfer 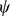 which is the fraction of transferred synapses which were correctly transferred. Both of these quantities depend on the stimulation fraction 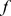 and the threshold 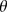 and can be calculated analytically, see *Methods*. In short, the stimulation of neurons during the transfer process leads to a unimodal input distribution which is approximately Gaussian for 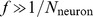. The transfer rate is proportional to the area in the tails of this distribution above the high threshold and below the low threshold, while the accuracy is the fraction of this area which is due only to strong synapses (above the high threshold) or to weak synapses (below the low threshold). It is easy to see that as the thresholds are moved away from the mean into the tails the transfer rate will decrease while the accuracy will increase. There is therefore a speed-accuracy tradeoff in the transfer process.

Additionally, the transfer process can be implemented even if we relax the assumption of strong one-to-one feedforward connections and allow for random feedforward projections, see *Text S1*. In this case a two-threshold rule is still needed to obtain performance above chance level, although an analytical description is no longer straightforward.

**Figure 6:**
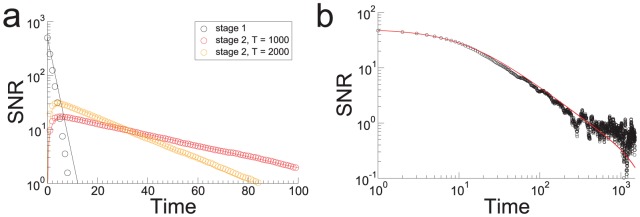
Memory consolidation in a neuronal model. **a.** The *effective* learning rate of a downstream synapse depends on the transfer process itself. Increasing the number of transfer repetitions 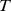 increases this rate leading to faster learning and faster forgetting. Shown is SNR of each of the first two stages. Symbols are averages of ten simulations, lines are from the mean-field model, see *Methods*. Here 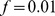, 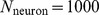, 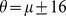, and 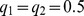 which gives 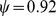 and 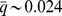 when 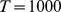. **b.** The neuronal model is well described by the mean-field synaptic model. There are 10 stages, each with 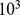 all-to-all connected neurons. Parameters are chosen such that transfer rates are 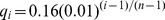. The solid line is for a 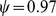 in the mean-field model. Shown is the combined SNR for all 10 stages.

The neuronal implementation of the transfer process reveals an important fact: the probability of correctly updating a synapse does not depend solely on its intrinsic learning rate, but rather on the details of the transfer process itself. In our simple model, the transfer rate is 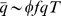 where 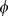 is a factor which depends on the threshold of the learning process relative to the distribution of inputs and 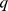 is the intrinsic learning rate of the synapses in the downstream stage. Additionally, since the likelihood of a correct transfer is 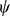, the rate of correct transfers is 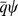, while there is also a “corruption” rate equal to 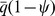 which is the probability of an incorrect transfer. Obviously, if a given fraction of synapses is to be transferred correctly, the best strategy is to make 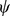 as close to one as possible and increase 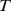 accordingly. In the limit 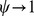 the neuronal model is exactly equivalent to the mean-field model we studied earlier with the transfer rate 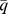 playing the role of the learning rate. For 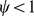 a modified mean-field model with a “corruption” term can be derived, see *Text S1* for details. Fig. 6 illustrates that the neuronal implementation quantitatively reproduces the behavior of the synaptic mean-field model. Specifically, the transfer rate can be modified by changing the number of transfers 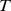, as shown in Fig. 6a. In this case, although the intrinsic synaptic properties have not changed at all, learning and forgetting occur twice as fast if 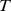 is doubled. The combined SNR of ten stages with 1000 all-to-all connected neurons each averaged over ten realizations (symbols) is compared to the mean-field model (line) in Fig. 6. In this case, the parameters of the neuronal model have been chosen such that the transfer rates are equal to 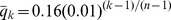, and 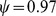.

## Discussion

In conclusion, we showed that there is a clear computational advantage in partitioning a memory system into distinct stages, and in transferring memories from fast to slow stages. Memory lifetimes are extended by a factor that is proportional to the number of stages, without sacrificing the amount of information stored per memory. For the same memory lifetimes, the initial memory strength can be orders of magnitude larger than in non-partitioned homogeneous memory systems. In the Results we focused on the differences between the heterogeneous and the memory system model. In Fig. S15 in *Text S1* we show that the SNR of the memory transfer model (multistage model) is always larger than the SNR of homogeneous model for any learning rate. This is true also when one considers that homogeneous models can potentially store more information than the memory transfer model. Indeed, in the homogeneous model all synapses can be modified at the time of memory storage, not only the synapses of the first stage. However, the main limitation of homogeneous models with extended memory lifetimes comes from the tiny initial SNR. If one reduces the amount of information stored per memory to match the information stored in the memory transfer model, it is possible to extend an already long memory lifetime but the initial SNR reduces even further (see *Text S1* for more details).

Our result complements previous studies (see e.g. [8], [26], [27]) on memory consolidation that show the importance of partitioning memory systems when new semantic memories are inserted into a body of knowledge. Two-stage memory models were shown to be fundamentally important to avoid catastrophic forgetting. These studies focused mostly on “memory reorganization”, as they presuppose that the memories are highly organized and correlated. We have solved a different class of problems that plague realistic memory models even when all the problems related to memory reorganization were solved. The problems are related to the storage of the memory component that contains only incompressible information, as in the case of random and uncorrelated memories. These problems are not related to the structure of the memories and to their similarity with previously stored information, but rather they arise from the assumption that synaptic efficacies vary in a limited range. We showed here that this problem, discovered two decades ago [3] and partially solved by metaplasticity [11], can also be solved efficiently at the systems level by transferring memories from one sub-system to another.

Our neuronal model provides a novel interpretation of replay activity. Indeed, we showed that in order to improve memory performance, synapses should be copied from one stage to another. The copying process occurs via the generation of neuronal activity, that reflects the structure of the recurrent synaptic connections to be copied. The synaptic structure, and hence the neuronal activity, is actually correlated with all past memories, although most strongly with recent ones. Therefore while this activity could be mistaken for passive replay of an individual memory, it actually provides a snapshot of all the information contained in the upstream memory stage. There is already experimental evidence that replay activity is not a mere passive replay [28]. Our interpretation also implies that the statistics of “replay” activity should change more quickly in fast learning stages like the medial temporal lobe, than in slow learning stages like pre-frontal cortex or some other areas of the cortex [18].

Our analysis also reveals a speed-accuracy trade off that is likely to be shared by a large class of neuronal models that implement memory transfer: the faster the memories are transferred (i.e. when a large number of synapses are transferred per “replay” and hence a small number of repetitions 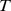 is needed), the higher the error in the process of synaptic copying (Fig. 6a). Accuracy is achieved only when the number of synapses transferred per “replay” is small and 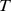 is sufficiently large. This consideration leads to a few requirements that seem to be met by biological systems. In particular, in order to have a large 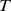, it is important that the transfer phases are brief, if the animal is performing a task. This implies that the synaptic mechanisms for modifying the synapses in the downstream stages should operate on short timescales, as in the case of Spike Timing Dependent Plasticity (STDP) (see e.g. [29]). Alternatively, the transfer can occur during prolonged intervals in which the memory system is off-line and does not receive new stimuli (e.g. during sleep).

Although we have focused on the transfer of memories in our model, the neuronal model can additionally be used to read out memories. Specifically, the neuronal response of any stage (or several stages) to a previously encoded pattern is larger than to a novel pattern. This is true as long as the SNR, as we have used it in this paper i.e. synaptic overlap, is sufficiently large. This difference in neuronal response can be used by a read-out circuit to distinguish between learned and novel patterns, see *Text S1* for a detailed implementation.

Our theory led to two important results which generate testable predictions. The results are: 1) the memory performance increases linearly with the number of memory stages, and 2) the memory trace should vary in a non-monotonic fashion in most of the memory stages. The first suggests that long-term memory systems are likely to be more structured than previously thought, although we cannot estimate here what the number of partitions should be, given the simplicity of the model. Some degree of partitioning has already been observed: for example graded retrograde amnesia extends over one or two years in humans with damage to area CA1 of the hippocampus, but can extend to over a decade if the entire hippocampus is damaged [30]. Systematic lesion studies in animals should reveal further partitioning in the hippocampal-cortical pathway for consolidation of episodic memories.

A second prediction is related, since once the partitions have been identified, our work suggests that most stages should exhibit non-monotonic memory traces, although on different time-scales. In fact, a recent imaging study with humans revealed non-monotonic BOLD activation as a function of the age of memories that subjects recalled [31]. Furthermore the non-monotonicity was observed only in cortical areas and not in hippocampus. Here multi-unit electrophysiology in animals would be desirable to obtain high signal-to-noise ratios for measuring the memory traces. An analysis such as the one proposed by [32], [33], in which spiking activity in rats during sleep was correlated with waking activity, should enable us to estimate the strength of a memory trace. We expect that the memory trace is a non-monotonic function of time in most memory areas. The initial trace is usually small or zero, it then increases because of the information transferred from the upstream memory stages, and it finally decreases as a consequence of the acquisition of new memories. The timescales of the rising phase should reflect the dynamics of the upstream memory stages, whereas the decay is more related to the inherent dynamical properties of the memory stage under consideration. Therefore, the position of the peak of the memory trace and the timescale of the decay give important indications on the position of the neural circuit in the memory stream and on the distribution of parameters for the different memory stages. The statistics of neural activity during memory transfer (replay activity) should reflect the synaptic connections and in particular it should contain a superposition of a few memory traces in the fast systems, and an increasingly larger number of traces in the slower systems. The statistics of the correlations with different memories should change rapidly in the fast systems, and more slowly in the slow systems (e.g. in the hippocampus the changes between two consecutive sleeping sessions should be larger than in cortical areas where longer-term memories are stored).

To obtain experimental evidence for these two sets of predictions, it is important to record neural activity for prolonged times, in general long enough to cover all the timescales of the neural and synaptic processes that characterize a particular brain area. This is important both to determine the time development of the memory traces and to understand the details of the neural dynamics responsible for memory transfer.

To estimate the SNR, one can analyze the recorded spike trains during rest and NREM sleep, when memory transfer is expected to occur. We believe that the strength of memory reactivation is related to our SNR. The analysis proposed in [32], [33] should allow us to estimate the templates of memories that are reactivated during one particular epoch (the templates are the eigenvectors of the covariance matrix that contains the correlations between the firing rates of different neurons). The time development of the memory trace can be then studied by projecting the activity of a different epoch on the eigenvectors. The projections are a measure of the memory reactivation strength and they should be approximately a nonlinear monotonic function of the memory signal. This analysis not only would determine whether the memory trace is a non-monotonic function of time but it would also allow us to estimate the parameters that characterize its shape in different brain areas.

The memory model studied here is a simple abstraction of complex biological systems which illustrates important general principles. Among the numerous simplifications that we made, there are three that deserve additional discussion. The first one is about the representations of the random memories and the second one is about the synaptic dynamics.

The first simplification is that we implicitly assumed that the memory representations are dense, as all synapses are potentially modified every time a new memory is stored. In the brain these representations are likely to be sparse, especially in the early stages of the memory transfer model, which probably correspond to areas in the medial temporal lobe. Sparseness is known to be important for increasing memory capacity [3], [34], [35] and one may legitimately wonder why we did not consider more realistic sparse representations. However, in our simplified model sparser random representations are equivalent to lower learning rates if the average number of potentiations and depressions are kept balanced. If 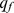 is the average fraction of synapses that are modified in the first stage (coding level), then all *q*s of the model should be scaled by the same factor 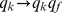. This does not change the scaling properties that we studied, except for a simple rescaling of times (the x-axis of the plots should be transformed as 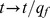) and SNR (SNR→SNR·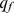). In conclusion, sparseness is certainly an important factor and we are sure that it plays a role in the memory consolidation processes of the biological brain. However here we focused on mechanisms that are independent from the coding level and hence we did not discuss in detail the effects of sparseness, which have been extensively studied elsewhere [3], [34], [35].

The second simplification that merits a further discussion is that the model synapses studied here have a single time-scale associated with each of them. Our model can be extended to include synaptic complexity as in [11]. In fact, allowing for multiple time-scales at the level of the single synapse should lessen the number of stages needed for a given level of performance. Specifically, time-scales spanning the several orders of magnitude needed for high SNR and long memory lifetimes can be achieved through a combination of consolidation processes both at the single synapse, and between spatially distinct brain areas.

## Methods

Here we include a brief description of the models and formulas used to generate the figures. For a detailed and comprehensive description of the models please refer to *Text S1*.

### Simple models of synaptic memory storage

The homogeneous and heterogeneous synaptic models are comprised of 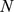 stochastically updated binary synapses which evolve in discrete time. In the homogeneous case all synapses have the same learning rate 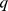, while in the latter case there are 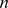 groups of 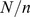 synapses each. Each group 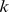 has a learning rate 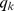. At each time step all 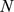 synapses are subjected to a potentiation or depression with equal probability. The N-bit word of potentiations and depressions constitutes the memory to be encoded. The memory signal at time 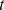, 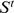 is the correlation of the 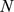 synaptic states with a particular N-bit memory, and we use superscript 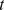 to denote evolution in discrete time. The signal-to-noise ratio (SNR) is approximately (and is bounded below by) the signal divided by 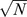, see *Text S1* for more details.

To compare with these Markov models one can derive a mean-field description which captures the memory signal averaged over many realizations of the stochastic dynamics. This is done by considering the probability that a given synapse is in a given state as a function of time. Specifically, the probability of a single synapse with learning rate 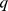 to be in the potentiated state at time 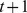 is just
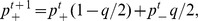
where 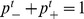 and 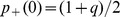.

In the case of the homogeneous synaptic model there are 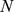 synapses with the same learning rate. The expected value of the signal averaged over realizations is then
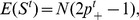
and so the expected signal-to-noise ratio is
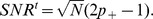
We can approximate the finite-time equation for 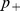 with a continuous ordinary differential equation which, using the definition of SNR gives
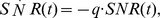


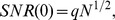
the solution of which is 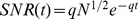. This equation is used to plot the curves in Fig. 1b. The heterogeneous case is analogous with
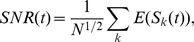


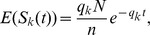
where 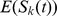 is the expected signal at time 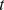 in stage 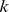. This equation is used to plot the solid curve in Fig. 1c. The SNR in the heterogeneous model can be increased by reading out only some of the groups at any one point in time, as opposed to all of them. This optimal readout is used to plot the dashed curves in the top panel of Fig. 3.

### The memory transfer model

Once again we assume there are a total of 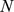 synapses divided equally amongst 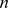 stages. Synapses in stage 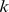 have learning rate 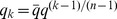 and hence the fastest learning rate is 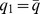 and slowest is 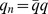. Synapses in stage 1 are updated every time step in an identical fashion to those in group 1 of the heterogeneous model above. Synapses in downstream stages however, update according to the state of counterpart synapses in the upstream stage. Specifically, if a synapse 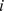 in stage 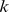 is potentiated (depressed) at time 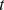, then synapse 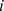 in stage 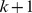 potentiates (depresses) at time 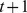 with probability 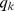. As before, the signal at time 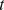 in stage 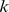 is written 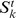. This fully defines the stochastic model.

As before we can derive a mean-field description of the stochastic dynamics. In this case, the probability of a given synapse in stage 1 to be in a potentiated state at time 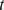 is 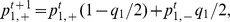


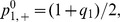
as in the simple models. The probability of a given synapse in stage 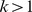 begin in a potentiated state can be written
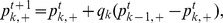


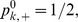
see *Text S1* for details. These equations reflect the fact that only synapses in stage 1 are updated due to the presentation of random, uncorrelated memories, while synapses in downstream stages are updated only due to the state of synapses in the preceding stage. The expected signal in stage 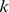 is given by 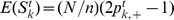.

The continuous time approximation to the mean-field dynamics is given by the set of equations
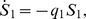


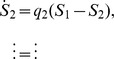


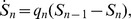
with initial conditions 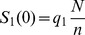, 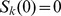 for 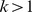 and we write 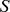 for the expected signal. These equations are used to plot the curves in Fig. 2a and the solid curves in the top panel of Fig. 3. For 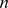 sufficiently large we can furthermore recast this system of ODEs as a PDE
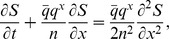


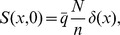
where the spatial variable 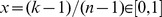. An asymptotic solution to this equation valid for 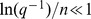, and taking now the SNR, is
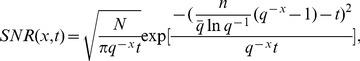
(1)see *Text S1* for details. This equation is used to plot the pulse solution shown in Fig. 2b. An optimal SNR, in which only some of the stages are read out, can be calculated based on Eq. 1 and is
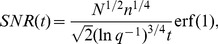
(2)which is valid for intermediate times where the SNR is powerlaw in form. This equation is used to plot the curves in Fig. 3 bottom left. Using Eqs. 1 and 2 one can calculate the lifetime of memories as
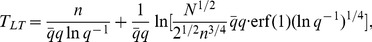
(3)if the SNR of the pulse is above one before reaching the last stage or
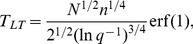
(4)is the SNR drops below one already before reaching the last stage. Eqs. 3 and 4 are used to plot the solid curves in Fig. 3 bottom right.

### Neuronal implementation of the memory transfer model

There are 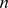 stages. Each stage is made up of 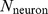 all-to-all coupled McCulloch-Pitts neurons. Each one of the 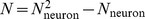 synapses (no self-coupling) can take on one of two non-zero values. Specifically, the synapse from neuron 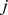 to neuron 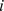 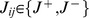, where 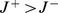. Furthermore, there are one-to-one connections from a neuron 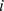 in stage 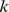 to a neuron 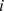 in stage 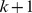. The model operates in two distinct modes: Encoding and Transfer.

#### Encoding

All memories are encoded only in stage 1. Specifically, one half of the neurons are randomly chosen to be activated (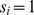 if 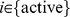), while the remaining neurons are inactive (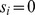 if 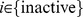). A synapse 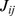 is then potentiated to 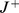 with a probability 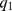 if 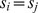 and is depressed with probability 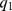 if 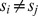.

#### Transfer

A fraction 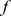 of randomly chosen neurons in stage 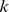 is activated at time 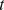. Because of the powerful feedforward connections, the same subset of neurons is activated in stage 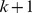. The recurrent connectivity may lead to postsynaptic activation in stage 1 neurons. Each neuron 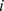 receives an input
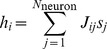
at time 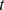 where 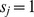 if neuron 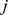 was activated and 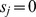 otherwise. The input 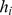 is a random variable which for 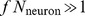 is approximately Gaussian distributed with expected mean and variance
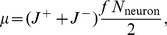


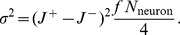
If 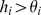, where 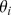 is the neuronal threshold, then neuron 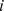 is activated at time 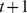. Again, because of the powerful feedforward connections, the same subset of neurons in stage 2 is activated. We take 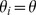 to be the same for all neurons and assume that it can take one of two values 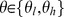 with equal likelihood during each replay.

For a transfer process with 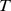 stimulations of a fraction 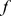 of neurons, the fraction of synapses updated in the downstream stage, or the transfer rate 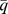, is a function of the area of the input distribution above (below) 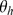 (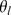). If the thresholds are placed equidistant from the mean 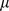, then
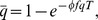


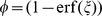


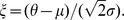
If the fraction of synapses transferred is small then 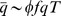, which is the formula given in the text. Of those synapses which are updated, only some will be updated correctly. This is equal to the fraction of potentiated (depressed) synapses contributing to the total input above (below) 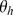 (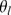), and is
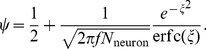
(5)Finally, the mean-field model describing the memory signal in each stage in the neuronal model is the same as in Eqs. 1-1 where the learning rate 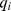 is now the transfer rate times the fraction of correct transfers 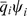, and there is an additional decay term due to incorrect transfers of the form 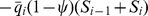 for 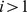. This mean-field model is used to make the solid curves in Fig. 6, whereas the symbols are from the full, Markov model with McCulloch-Pitts neurons.

## Supporting Information

Text S1 Additional model information.
(PDF)
