# Supplementary material for: Efficient Partitioning of Memory Systems and Its Importance for Memory Consolidation
Source: PLoS Comput Biol. 2013 Jul 25;9(7):e1003146. doi: 10.1371/journal.pcbi.1003146 (PMC3723499; doi:10.1371/journal.pcbi.1003146)
Supplement: Text S1 — Additional model information. (PDF) [file pcbi.1003146.s001.pdf]

Text S1

Contents

1 Brief Comment on Models 2

2 A synaptic model with homogeneous transition probabilities. (Fig.1a and b) 3

2.1 The Markov model 3

2.2 The continuous-time approximation 4

3 A synaptic model with heterogeneous transition probabilities. (Fig.1c) 5

3.1 The Markov model 5

3.2 The continuous-time approximation 6

3.3 Optimal readout of the memory signal 7

3.4 Summary 7

4 The synaptic memory transfer model (Figs.2 and 3) 7

4.1 The Markov model 9

4.2 Readout of the memory signal: the role of correlations 18

4.2.1 Correlations in a naive readout 20

4.2.2 Correlations in a readout of only a subset of stages . . 22

4.3 Naive readout of the memory signal 22

4.4 Optimal readout of the memory signal 22

4.5 A plausible read-out which is nearly optimal 29

4.6 The performance of the memory transfer model: Crossing times, SNR and memory lifetimes (Fig.3) 30

4.6.1 The crossing time T_c 31

4.6.2 The SNR in the powerlaw regime 31

4.6.3 Memory lifetime 31

4.7 Comparison between the multi-stage memory transfer model and homogenous (single stage) models 33

5 The neuronal memory transfer model (Fig.3) 39

5.1 The Markov model 39

5.1.1 Encoding 39

5.1.2 Transfer 40
5.1.3 The fraction of synapses transferred during replay . . . 41
5.1.4 The fraction of synapses correctly transferred during replay 43
5.2 Meanfield model 45
5.3 Build-up of correlations for many stages 46
5.4 Neuronal readout of memories 47

6 A neuronal memory transfer model with random projections 53
6.1 Initializing matrices 53
6.2 Calculating overlap 54
6.3 Replay 54

1 Brief Comment on Models

In this supplementary information we describe the models used in the paper in detail. The details of all relevant analysis are also included. We first discuss the homogeneous and heterogeneous models. Then there are two memory transfer models: 1 - the "synaptic model" in which the neuronal activity is not explicitly modeled, 2 - the "neuronal model" which does include neuronal activity. Both models are Markov models, i.e. they are stochastically updated in discrete time where the state of variables at a time $t+1$ depends only on the state of the variables at time t. We consider the memory capacity of these models by adopting an ideal observer approach. That is, we track the mnemonic trace of one particular memory, encoded in the pattern of synaptic weights of the model, until it is unrecoverable. For the models considered here we can obtain an analytical formula for the memory trace which allows us to determine how important measures such as the initial memory strength and memory lifetime, scale with the system size, i.e. how many synapses are available. All analytical results are obtained by studying continuous-time approximations to these Markov processes. These approximations take the form of ordinary and partial differential equations. We have organized this supplementary information in order to accompany the figures in the paper.

2 A synaptic model with homogeneous transition probabilities. (Fig.1a and b)

2.1 The Markov model

We consider binary synapses, which can be in one of two states: depressed or potentiated. At each time step, each synapse is independently presented with a plasticity event (see top left of Fig.1a) which can be either potentiating or depressing with probability 1/2. A depressed (potentiated) synapse presented with a potentiating (depressing) event will change state with a probability q, which is called the transition probability. The binary nature of the synapses means that only one memory may be encoded at a time. This is illustrated in Fig.1a by a color code. In the model we consider the state of N synapses simultaneously, and the 'memories' are therefore the combined N plasticity events to which the synapses are subjected, see bottom left of Fig.1a. These memories are random and uncorrelated from one time step to the next. As time progresses, we keep track of how similar the state of the N synapses is to one particular memory. Since all memories are identically distributed, tracking one is equivalent to tracking any other one and will tell us how all memories decay in time.

We can formalize this model description by assigning the value 1 to a potentiated synapse and -1 to a depressed one. Similarly, a plasticity event is assigned a value 1 if it is potentiating and -1 if depressing. We then define a vector of length N, $\mathbf{J}^t$ where $J_i^t \in \{-1, 1\}$ is the state of synapse i at time t. Similarly, the memories are also vectors of length N, $\mathbf{m}^t$, where $m_i^t \in \{-1, 1\}$ is the plasticity event to which synapse i is subjected at time t. If we choose to track the memory presented at time t^*, then we define the memory trace as the signal at time t, which is just the dot product of two vectors, $S^{t-t^*} = \mathbf{m}^{t^*} \cdot \mathbf{J}^t$. The signal itself is a stochastic variable, since the updating of the synaptic states is stochastic. This means that if one runs several simulations presenting exactly the same memories, the signal will be different each time, see right hand side of Fig.1a. The mean signal, understood as the signal averaged over many realizations of the Markov process, can be computed analytically. We compare this signal to the background noise which is defined as the standard deviation in the overlap between uncorrelated memories.

The probability of finding a synapse in a potentiated state at time $t + 1$

is

$$p_+^{t+1} = p_+^t(1 - q/2) + p_-^t q/2, \tag{S.1}$$

where $p_+^t = \Pr(J^t = 1)$. Using the fact that $p_+^t + p_-^t = 1$ allows one to write

$$p_+^{t+1} - p_+^t = q(1/2 - p_+^t). \tag{S.2}$$

In tracking one particular memory, we wish to know p_+^t for those synapses which were subjected to a potentiation when that memory was presented. Therefore, the initial condition is $p_+^0 = (1 + q)/2$. An identical equation and initial condition govern the dynamics of p_-.

The number of potentiated synapses n_+^t follows a Binomial distribution with probability p_+^t. It has mean $E(n_+) = \frac{N}{2}p_+^t$ and variance $var(n_+^t) = \frac{N}{4}p_+^t(1 - p_+^t)$. The mean signal or memory trace can be written

$$\begin{aligned} E(S^t) &= 2\big(E(n_+^t) - E(n_-^t)\big), \\ &= N\big(p_+^t - (1 - p_+^t)\big), \\ &= N\big(2p_+^t - 1\big). \end{aligned} \tag{S.3}$$

Note that the term -1 in the parentheses removes the trivial correlation (synapses are potentiated or depressed with probability 1/2 irrespective of the memory). The variance in the signal is $var(S^t) = 4Np_+^t(1-p_+^t)$. Writing $\bar{p}^t = 2p_+^t - 1$, we can then write the signal to noise ratio as $SNR^t = \sqrt{N}\frac{\bar{p}^t}{\sqrt{1-(\bar{p}^t)^2}}$, where $0 \leq \bar{p} \leq 1$. For simplicity, in this work we take $SNR^t = \sqrt{N}\bar{p}^t$, which is a lower bound for the true SNR and is the correct asymptotic expression for the SNR when $\bar{p}^t \ll 1$, e.g. at long times. This is so because at long times the probability of any synapse being potentiated is just one half, i.e. $p_+^t \to 1/2$ as $t \to \infty$, and $p_+^t = 1/2 + \bar{p}^t$.

While for the simple case of homogeneous synapses one can calculate the signal analytically in the discrete case ($S^t = q(1 - q)^t N$), solving for the signal in the continuous-time approximation is, in general, much easier. For the memory transfer model only the continuous-time approximation will yield analytical results. For this reason we consider now the continuous-time formulation of the Markov process.

2.2 The continuous-time approximation

A continuous-time approximation is made by assuming that the probability in Eq.S.2 changes little from one time step to the next. Then we replace the

difference on the left hand side by the time derivative to get

$$\dot{p} = q(1/2 - p), \tag{S.4}$$

with the initial condition $p(0) = (1+q)/2$ and we have dropped the subscript $+$. The mean signal can be written $\bar{S}(t) = N(2p(t) - 1)$ which leads to the equation

$$\dot{\bar{S}} = -q\bar{S}, \tag{S.5}$$
$$\bar{S}(0) = qN, \tag{S.6}$$

the solution of which is $\bar{S}(t) = qNe^{-qt}$, and the signal-to-noise ratio is therefore $SNR(t) = qN^{1/2}e^{-qt}$. In judging the performance of a model, we consider three salient characteristics of the SNR: 1 - the initial SNR, 2 - the functional form of the decay of the SNR, and 3 - the lifetime of the SNR. In the case of the homogeneous population of synapses, decay is exponential while the initial SNR is $qN^{1/2}$ and the lifetime, determined by setting the SNR equal to one, is $T = \frac{1}{q}\ln(qN^{1/2})$. It is clear that, for fixed N, taking a q near one will lead to a large initial SNR but a short lifetime, while a small q leads to a weak initial SNR but a long lifetime, see Fig.1b. This is a fundamental trade-off in populations of bounded synapses with homogeneous transition probabilities [2]. Significantly, adding synapses in order to increase memory lifetimes is extremely inefficient since the lifetime scales as $\ln N$.

3 A synaptic model with heterogeneous transition probabilities. (Fig.1c)

3.1 The Markov model

Each synapse is updated as in the homogeneous model with the difference that the transition probability is not the same for all synapses. Specifically, we consider n ensembles of N/n synapses such that the total number of synapses is N. Synapses in ensemble $k \in [1, n]$ have a transition probability $q_k = \bar{q}q^{(k-1)/(n-1)}$, so that synapses in ensemble 1 are the most plastic with a transition rate $\bar{q}$ and those in ensemble n are the least plastic with a transition rate $\bar{q}q$.

3.2 The continuous-time approximation

Following the same derivation as in the case of a population of homogeneous synapses leads to n distinct yet independent differential equations for the signal in each ensemble

$$\dot{\bar{S}}_k = -q_k\bar{S}_k, \tag{S.7}$$

$$\bar{S}_k(0) = q_k\frac{N}{n}, \tag{S.8}$$

the solution of which is $\bar{S}_k(t) = q_k\frac{N}{n}e^{-q_kt}$and the total signal is then $\bar{S}(t) = \sum_{k=1}^{n}\bar{S}_k(t)$. The initial SNR is given by

$$\begin{aligned} SNR(0) &= N^{1/2}\frac{1}{n}\sum_{k=1}^{n}q_k, && \text{(S.9)}\\ &= \bar{q}N^{1/2}\frac{1}{n}\frac{(1-q^{n/(n-1)})}{(1-q^{1/(n-1)})}, && \text{(S.10)}\\ &\sim \frac{\bar{q}}{\ln q^{-1}}N^{1/2}, && \text{(S.11)} \end{aligned}$$

where the last approximate formula holds as long as $\epsilon = \frac{1}{n-1}\ln q^{-1} \ll 1$. Thus, compared to the homogeneous model, the initial SNR is reduced by a factor proportional to the logarithm of the slowest transition rate $1/\ln q^{-1}$. The lifetime of the memory is determined by the SNR of the slowest ensemble and is $T = \frac{1}{q\bar{q}}\ln\left(q\bar{q}(N/n)^{1/2}\right)$, i.e. there is a weak reduction in the lifetime due to fact that more ensembles mean fewer synapses per ensemble, specifically the slowest ensemble. Interestingly, the functional form of the SNR is approximately powerlaw with exponent one, with an exponential cut-off, corresponding to the slowest ensemble. It is not hard to see how a sum of exponentials can generate a powerlaw dependence [1]. Consider the following sum of exponentials for which the exponent is itself exponentially distributed, as in the synaptic model $SNR(t) \sim N^{1/2}\int_q^1 dkf(k)e^{-\bar{q}kt} = N^{1/2}\int_q^1 dk\bar{q}e^{-\bar{q}k}e^{-\bar{q}kt} = N^{1/2}\frac{1}{1+t}\left(e^{-q\bar{q}(1+t)} - e^{-\bar{q}(1+t)}\right)$. In this continuum approximation it is clear that after an initial transient for times $t < 1/\bar{q}$, the decay is approximately powerlaw until a time $t > 1/(\bar{q}q)$, after which there is an exponential cutoff. In the intermediate regime $1/\bar{q} < t < 1/(\bar{q}q)$ the decay is approximately powerlaw with exponent one. In this regime, the lifetime scales as $N^{1/2}$ and not $\ln N$. Therefore, memory lifetimes can be significantly extended by adding more synapses.

3.3 Optimal readout of the memory signal

We have so far considered the case in which all synapses are read out simultaneously in order to determine the SNR of the memory. In the case of heterogeneous synapses, however, we could envisage an optimal readout by considering only certain ensembles of synapses so as to maximize the SNR. Specifically, we could read out only those synapses from ensembles k_f to k_s, i.e. $SNR_{\text{optimal}}(t) = \max_{k_s(t),k_f(t)\in\{1,n\}} SNR(t)$, where $SNR(t) = \frac{N^{1/2}}{(k_s(t)-k_f(t))^{1/2}} \sum_{k=k_f(t)}^{k_s(t)} S_k(t)$. In the case of heterogeneous ensembles of synapses, the optimal readout provides a small increase in SNR, as shown in Fig.S.1A. Furthermore, this increase is essentially independent of the total number of ensembles in the model. For heterogeneous synapses it is optimal, at intermediate times, to read out a large fraction of the total number of ensembles as shown in Fig.S.1B.

3.4 Summary

In summary, allowing for heterogeneity in the transition rates, which is tantamount to having some synapses be very plastic and others less so, leads to small reductions in the initial SNR and memory lifetime for fixed N compared to the homogeneous case. However, the heterogeneity provides a significant boost to the SNR for intermediate times. Specifically , in the powerlaw regime lifetimes scale as $N^{1/2}$. The optimal readout for heterogeneous synapses leads only to a small improvement compared to reading out all ensembles, and requires reading out a significant fraction of ensembles in the powerlaw regime.

4 The synaptic memory transfer model (Figs.2 and 3)

For clarity we first list here the main model parameters, variables and functions.

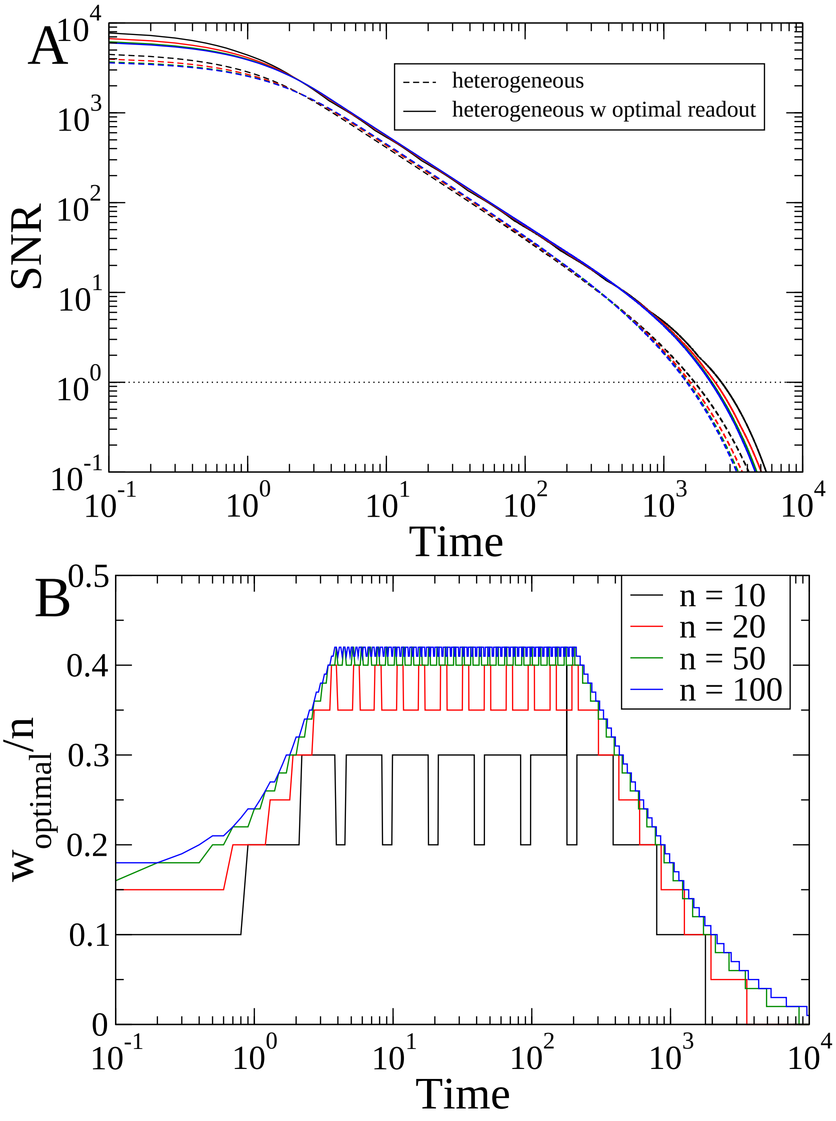

Fig. S.1: A. The SNR for heterogeneous ensembles of synapses both by reading out all ensembles (dashed lines) as well as using an optimal readout (solid lines). The total number of synapses is $N = 10^9$ which are divided into $n = 10, 20, 50$ and 100 ensembles (black, read, green, blue). Here $q_i = \bar{q}q^{(i-1)/(n-1)}$ with $\bar{q} = 0.8$ and $q = 0.001$. B. The width of the optimal readout scaled by the total number of ensembles of synapses.

Symbol	Description
N	Total number of synapses
n	Number of stages
$q_i = \bar{q}q^{(i-1)/(n-1)}$	Learning rate of stage i
$q_1 = \bar{q}$	Fastest learning rate
$q_n = \bar{q}q$	Slowest learning rate
S_i^t	Signal in stage i at time t in discrete-time model
SNR_i^t	Signal-to-noise ratio in stage i at time t in discrete-time model
$S_i(t)$	Signal in stage i at time t in continuous-time model
$SNR_i(t)$	Signal-to-noise ratio in stage i at time t in continuous-time model

4.1 The Markov model

In the memory transfer model synapses take on different transition probabilities, as in the heterogeneous model. However, unlike in the heterogeneous model, synapses from one ensemble in the consolidation model may affect the state of synapses in another ensemble. Specifically, N synapses are arranged into n stages of N/n synapses each, and the stages interact in a feedforward manner. Memories are encoded only in the state of synapses in stage 1. The states of synapses in stage 2 depend on the states of synapses in stage 1 and so on. Furthermore, the synapses in stage 1 are taken to be the most plastic and synapses in each stage thereafter are progressively less plastic. In this way we seek to capture a consolidation process by which memories are initially encoded with a strong SNR in the input stage and then are transferred into deeper stages where the SNR lifetimes become progressively longer. It was shown previously that such a scheme implemented via a cascade of metaplastic states at the level of a single synapse can greatly improve memory capacity over models of simple synapses [1]. Here we implement a similar idea in a *spatially* segregated model with feedforward structure.

Specifically, at time t, a memory $\mathbf{m}^t$ of length N/n consisting of a random pattern of potentiating ($m_i^t = 1$) and depressing ($m_i^t = -1$) events is presented to the N/n synapses in stage one, which have synaptic state $\mathbf{J}_1^t$. Synapse i is subjected either to a potentiating ($m_i^t = 1$) or to a depressing ($m_i^t = -1$) event with probability 1/2, and is updated with a probability q_1 as in the previous models. Therefore, the updating for synapses in stage 1 is identical to that for ensemble 1 in the synaptic model with heterogeneous transition probabilities in Section 3. We then assume that a synapse i in stage 2 is influenced by the state of synapse i in stage 1 in the following way.

If synapse i in stage 1 is in a potentiated (depressed) state at time t ($J_1^t = 1$ or $J_1^t = -1$ respectively), then synapse i in stage 2 will potentiate (depress) at time $t+1$ with probability q_2. The update rule for synapses in stage 3 proceeds analogously, but depends now on the state of synapses in stage 2, and so on. See Fig. 2a for a schematic of this update rule.

We can formalize the update rule mathematically as before. The probability of finding that a synapse in stage 1 is in the potentiated state at time $t+1$ is

$$p_{(1,+)}^{t+1} = p_{(1,+)}^{t} + q_1(1/2 - p_{(1,+)}^{t}), \tag{S.12}$$

which follows from Eq.S.2.

In order to derive the update rule for stage $k > 1$, we must take into consideration the fact that the probabilities in stage $k > 1$ are dependent on those in stage $k-1$. The probabilities may, in fact, be correlated. Therefore, the probability of finding that a synapse in stage 2 is in the potentiated state at time $t+1$ is

$$\begin{aligned} p_{(k,+)}^{t+1} &= p_{(k,k-1;+,+)}^{t} + p_{(k,k-1;+,-)}^{t}(1-q_k) + q_k p_{(k,k-1;-,+)}^{t}, \\ &= p_{(k,+)}^{t} + q_k\Big(p_{(k,k-1;-,+)}^{t} - p_{(k,k-1;+,-)}^{t}\Big), \\ &= p_{(k,+)}^{t} + q_k\Big(p_{(k,k-1;-,+)}^{t} + p_{(k,k-1;+,+)}^{t} - p_{(k,k-1;+,+)}^{t} - p_{(k,k-1;+,-)}^{t}\Big), \\ &= p_{(k,+)}^{t} + q_k\Big(p_{(k-1,+)}^{t} - p_{(k,+)}^{t}\Big), \end{aligned} \tag{S.13}$$

where $p_{(k,k-1;a,b)}$ is the joint probability distribution for a synapse in stage k to be in a state $a \in \{-,+\}$ and the corresponding synapse in stage $k-1$ to be in a state $b \in \{-,+\}$.

Again, because the number of potentiated and depressed synapses follow Binomial distributions, we can write $SNR_k^t = \sqrt{N}\frac{\bar{p}_k^t}{\sqrt{1-(\bar{p}_k^t)^2}}$, where $\bar{p}_k^t = 2p_{k,+}^t - 1$. We approximate this as $SNR_k^t = \sqrt{N}\bar{p}_k^t$ which is a lower bound on the true SNR and the correct asymptotic form of the SNR for $\bar{p}_k^t \ll 1$.

As before, these equations can be solved analytically, but become increasingly unwieldy for downstream stages. It is easier to do analysis in the continuous-time approximation.

The continuous-time approximation

In the continuous-time approximation, discrete time differences become time derivatives, and one has the following set of equations

$$\dot{S}_1 = -q_1 S_1, \tag{S.14}$$
$$\dot{S}_2 = q_2(S_1 - S_2), \tag{S.15}$$
$$\vdots = \vdots \tag{S.16}$$
$$\dot{S}_n = q_n(S_{n-1} - S_n), \tag{S.17}$$

together with the initial condition

$$S_1(0) = q_1 \frac{N}{n}, \tag{S.18}$$
$$S_2(0) = 0, \tag{S.19}$$
$$\vdots = \vdots \tag{S.20}$$
$$S_n(0) = 0, \tag{S.21}$$

where as before $S_i(t) = \frac{N}{n}(2p_i(t)-1)$ and $q_{i+1} < q_i$. At this point, we consider how Eqs.S.14-S.21 reflect the phenomenon of consolidation. At time $t = 0$ a new 'memory' encoded in stage 1. This memory trace drives an increase in the memory trace in stage 2, which will then decay at a rate q_2, and so on. Therefore, while in the heterogeneous model the memory trace in stages 1 and 2 both decrease monotonically in time, in the memory transfer model, the memory trace in stage 2 is non-monotonic. It first increases, and then decreases, as shown in Fig.2b. At this point we can ask if the memory transfer model outperforms the benchmark heterogeneous model, even in the simplest case of two stages (or ensembles in the heterogeneous case). First we note that $S_1^h = S_1^{mt} = q_1 \frac{N}{n} e^{-q_1 t}$ is identical for both the heterogeneous (h) and the memory transfer (mt) models. On the other hand, $S_2^h = q_2 \frac{N}{n} e^{-q_2 t}$, while $S_2^{mt} = \frac{q_1 q_2}{q_1 - q_2} \frac{N}{n}(e^{-q_2 t} - e^{-q_1 t})$. These curves intersect at a time $T = \frac{1}{q_1 - q_2} \ln(q_1/q_2)$, and for times greater than T, i.e. $t = T + t'$ one finds that $S_1^{mt}/S_1^h = \frac{q_1 - q_2 e^{-(q_1-q_2)t'}}{q_1 - q_2}$, which is always greater than 1. Therefore, after a time T the memory transfer model will always outperform the heterogeneous model. In fact, we will show that improvement is largest when there are many stages, and the difference between transition probabilities in adjacent stages is small. If this is the case, we can write $q_2 = q_1 - \Delta q$ where $\Delta q \ll 1$ and

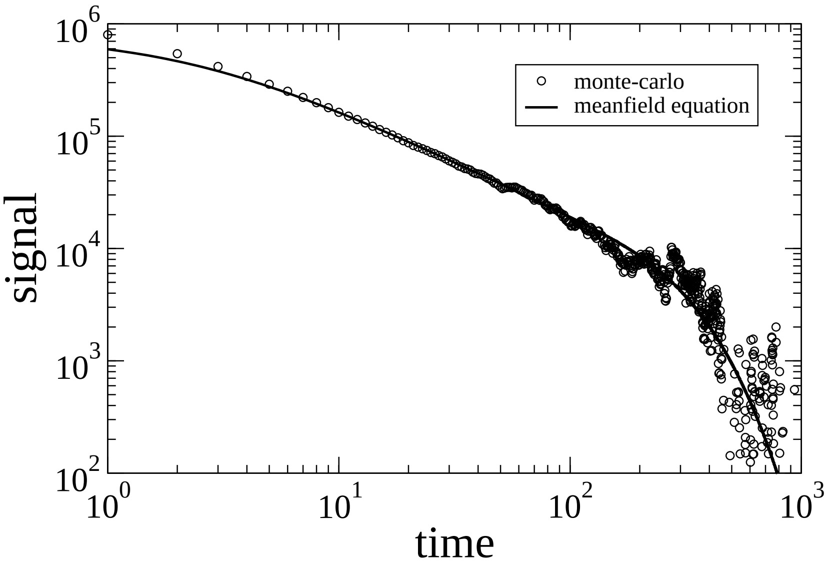

Fig. S.2: Comparison of the stochastic Markov model (black circles) and the continuous-time meanfield model (black line). See text for parameter values.

we find that $T \sim 1/q_1$ to leading order. Since q_1 is close to one (very plastic synapses in stage one) the improvement in performance of the consolidation model over the heterogeneous model comes about already at very short times.

Testing the meanfield model against the full Markov model

In order to compare the the meanfield model with the full Markov model we conduct Monte-Carlo simulations of the stochastic Markov model given binary synapses with a learning rate in stage i of $q_i = 0.8 * (0.01)^{(i-1)/9}$ for a total of $n = 10$ stages. Each stage has 10^6 synapses for a total of 10^7 synapses. The total signal averaged over 10 runs is shown as black circles in Fig.S.2. With this we compare the a simulation of the continuous-time meanfield model Eqs.S.14-S.17 (black line). Clearly, the meanfield model captures the ensemble average of the Monte-Carlo simulations quite well.

The curve plotted in Fig.S.2 is the total signal, summed over all stages. We can also look at the signal in each stage individually. This is shown in Fig.S.3 for stages 1,2,4 and 7 out of ten. Not all stages are shown for the sake of clarity. Here it is clear that there is a discrepancy between the

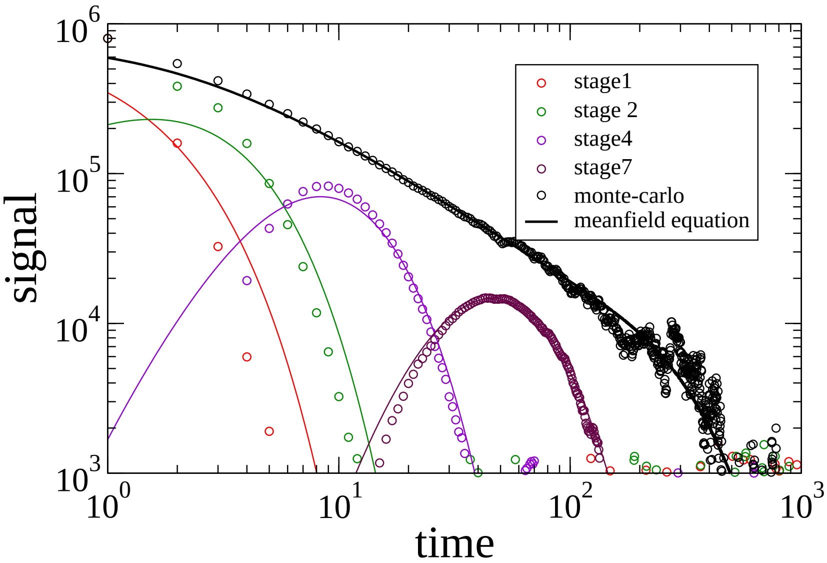

Fig. S.3: Comparison of the stochastic Markov model (circles) and the continuous-time meanfield model (lines). See text for parameter values.

continuous-time meanfield model and the full Markov model at short times (and hence in early stages). This discrepancy is due to the assumption of evolution in continuous time, whereas the Markov model evolves in discrete time. This can be seen by comparing the Markov model to the discrete-time meanfield model. In particular, we have solved the discrete-time meanfield model analytically for the first two stages. The solution for stage 1 is simply $S_1^t = \frac{N}{n} q_1 (1-q_1)^t$, whereas for stage 2, after a lengthy calculation (details not shown), one arrives at

$$S_2^t = \begin{cases} 0, & \text{if } t = 0 \\ \left(q_1(1-q_1)^{t-1} - q_1(1-q_2)^t + q_1^2 \sum_{k=1}^{t-1} (1-q_1)^{t-1-k}(1-q_2)^t\right)\frac{N}{n} & \text{if t} > 0. \end{cases} \tag{S.22}$$

The signal in downstream stages can be calculated but the formulae become increasingly unwieldy.

The result is shown in Fig.S.4, where the result from the discrete-time meanfield model is shown as squares. Clearly the agreement with the stochastic Markov model is excellent.

Finally, Fig.S.5 shows the total signal, averaged over all 10 stages, for all 10 simulations of the stochastic Markov model. Two of the signals are shown

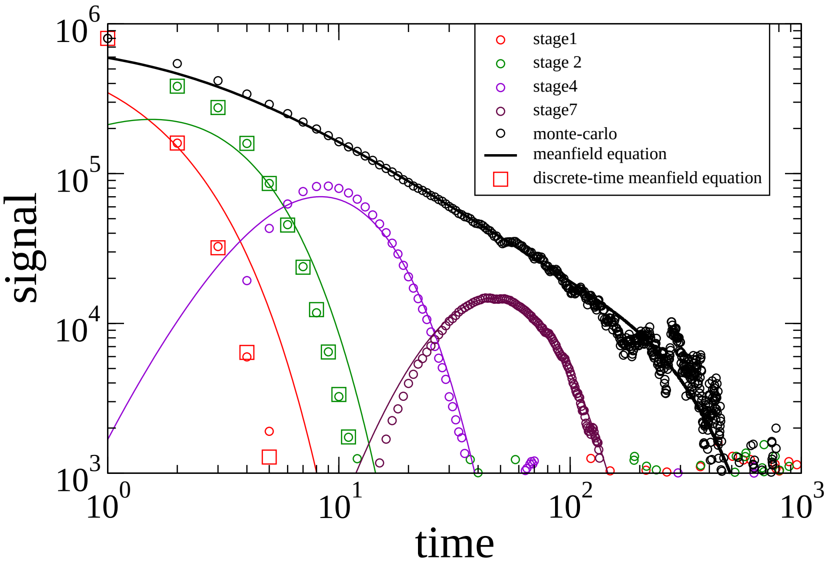

Fig. S.4: Comparison of the stochastic Markov model (circles), continuous-time meanfield model (lines) and discrete-time meanfield model (squares). See text for parameter values.

as solid lines as examples. We also plot the result of the meanfield model (solid green line). We define the SNR as the signal divided by $N^{1/2}$ which, as we show above, is a lower bound on the SNR and is a good approximation for stage k as long as $p_k \ll 1$. It is clear that the fluctuations in the signal indeed are as large as the signal itself on a single run once the SNR approaches 1.

The continuous-time continuous-space approximation

If the number of stages is large enough we can recast Eqs.S.14-S.21 in the form of a partial differential equation. In doing so, we are thinking of the index of the stages as a spatial variable and assuming that nearby stages have similar signals. Specifically, we write $S_i(t) \sim S(x,t)$, $q_i \sim q(x)$ and then expand $S_{i-1}(t) \sim S(x-dx,t) = S(x,t) - dx\frac{\partial S(x,t)}{\partial x} + dx^2 \frac{1}{2}\frac{\partial^2 S(x,t)}{\partial x^2} + \ldots$, where $dx = 1/n$ (and so $x \in [0,1]$). Then Eqs.S.14-S.21 become

$$\frac{\partial S}{\partial t} + \frac{\bar{q}q^x}{n}\frac{\partial S}{\partial x} = \frac{\bar{q}q^x}{2n^2}\frac{\partial^2 S}{\partial x^2}, \quad \text{(S.23)}$$

$$S(x,0) = \bar{q}\frac{N}{n}\delta(x), \quad \text{(S.24)}$$

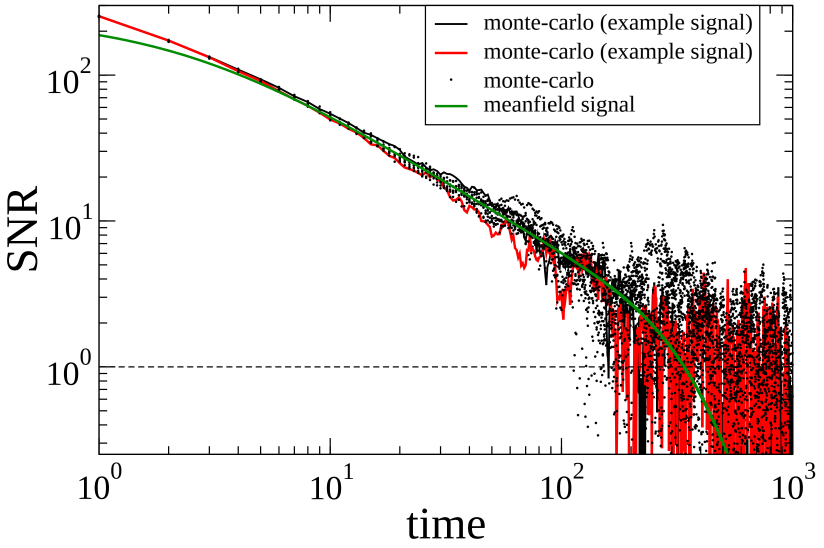

Fig. S.5: Comparison of total signal from all ten simulations of the Markov model with the continuous-time meanfield model. In this case, we define the SNR as begin equal to the signal divided by $N^{1/2}$. This is a lower bound on the SNR as described above. Two sample signals from the Markov model are shown as a solid lines. See text for parameter values.

where $\delta(x)$ is the Dirac delta function and we have dropped all terms of order $1/n^3$ and higher. Furthermore, we choose $q(x) = \bar{q}q^x$ to be consistent with the Markov model. Eqs.S.23-S.24 represent an advection diffusion process in which the initial condition is a pulse of amplitude $\bar{q}N/n$ at $x = 0$. The pulse travels to the right and slowly diffuses, eventually exiting the system at $x = 1$. Moreover, both the velocity of the pulse and the diffusion depend on the spatial location of the pulse, i.e. they are not homogeneous. Note that the pulse represents the correlation of the synaptic weights with a particular memory. Therefore, it is this correlation, i.e. the 'memory' which is propagating.

The advection equation

We can determine the spatial dependence of the pulse velocity by ignoring the diffusion term and solving

$$\frac{\partial S}{\partial t} + \frac{\bar{q}q^x}{n}\frac{\partial S}{\partial x} = 0, \qquad \text{(S.25)}$$

$$S(x,0) = \bar{q}\frac{N}{n}\delta(x),, \qquad \text{(S.26)}$$

which is a pure advection equation. In this equation the initial pulse propagates to the right without changing shape. The curve along which it travels in space and time $c(x,t)$ can be found by the so-called method of characteristics. Since the signal pulse doesn't change along this curve we can write

$$\frac{\partial S}{\partial c} = \frac{\partial S}{\partial t}\frac{\partial t}{\partial c} + \frac{\partial S}{\partial x}\frac{\partial x}{\partial c} = 0, \qquad \text{(S.27)}$$

which, comparing to Eq.S.26 means that $\frac{\partial c}{\partial t} = 1$ and $\frac{\partial c}{\partial x} = \frac{n}{\bar{q}}q^{-x}$. From this we find that $\frac{\partial x}{\partial t} = \frac{\bar{q}}{n}q^x$, which when integrated with the initial condition that $x(0) = 0$ gives the curve

$$t = \frac{n}{\bar{q}\ln q^{-1}}(q^{-x} - 1). \qquad \text{(S.28)}$$

For advection or wave phenomena where the velocity of propagation is constant, the characteristics have the form $x - vt$. Here it is clear that the propagation is not at a constant speed, but rather slows down exponentially. The memory lifetime is just the time at which the memory pulse exits the system at $x = 1$, i.e.

$$T \sim \frac{n}{\bar{q}q\ln q^{-1}}. \qquad \text{(S.29)}$$

From this we can see that the memory lifetime is boosted by a factor $n/\ln q^{-1}$ compared to the heterogeneous case (in the heterogeneous case the power law regime ends for $t \sim 1/(\bar{q}q)$, see Section 3.2). Conspicuously missing is the scaling with the number of synapses N. This is because we have not actually solved the true advection diffusion equation. For the pure advection equation, the amplitude of the pulse does not change in time as long as the initial SNR is above one, nothing is gained by adding more synapses. Once we include diffusion this will change.

The advection-diffusion equation

We can use the small parameter $\ln q^{-1}/n$ to find an approximate solution to the full equations, Eqs.S.23-S.24. Before doing this, we will perform a change of variables to eliminate the spatially variable velocity, i.e. we will stretch space so that the characteristics are just straight lines. We already know the correct change of variables from the preceding section. Specifically, we define the new spatial variable $y = \frac{n}{\bar{q}\ln q^{-1}}(q^{-x} - 1)$ and rewrite the PDE in terms of y. This requires using the chain rule for differentiation. The first and second derivatives are

$$\begin{aligned} \frac{\partial}{\partial x} &= \frac{\partial}{\partial y}\frac{dy}{dx}, \\ &= \frac{n}{\bar{q}q^x}\frac{\partial}{\partial y}, \end{aligned} \tag{S.30}$$

and

$$\begin{aligned} \frac{\partial^2}{\partial x^2} &= \frac{\partial}{\partial x}\Big(\frac{n}{\bar{q}q^x}\frac{\partial}{\partial y}\Big), \\ &= \frac{n\ln q^{-1}}{\bar{q}q^x}\frac{\partial}{\partial y} + \frac{n^2}{\bar{q}^2 q^{2x}}\frac{\partial^2}{\partial y^2}. \end{aligned} \tag{S.31}$$

The PDE becomes

$$\frac{\partial S}{\partial t} + \Big(1 - \frac{\epsilon}{2}\Big)\frac{\partial S}{\partial y} = \frac{1}{2\bar{q}}\Big(1 + \bar{q}\epsilon y\Big)\frac{\partial^2 S}{\partial y^2}, \tag{S.32}$$

$$S(y,0) = \bar{q}\frac{N}{n}\delta(y), \tag{S.33}$$

where $\epsilon = \ln q^{-1}/n \ll 1$. Since ϵ is small, we can ignore the small correction to the (now constant) velocity, but we cannot ignore the term proportional

to y in the diffusion. The reason is that y itself ranges between 0 and values of order $1/\epsilon$. Rather, we will define a new spatial variable $Y = \epsilon y$. Which allows us to write

$$\frac{\partial S}{\partial t} + \frac{\partial S}{\partial y} = \frac{1}{2\bar{q}}\Big(1 + \bar{q}Y\Big)\frac{\partial^2 S}{\partial y^2}, \tag{S.34}$$

$$S(y, 0) = \bar{q}\frac{N}{n}\delta(y), \tag{S.35}$$

The separation of variables y and Y has a physical interpretation here. The memory pulse propagates through the system, and as it does so, it slowly spreads out. The shape of the pulse depends on this diffusion, i.e. the second spatial derivative, which itself depends on the location of the pulse. However, the diffusion coefficient changes over a length scale which is large compared to the pulse shape itself. Therefore, we can treat the system as if the diffusion coefficient were locally constant. The constant coefficient advection-diffusion equation with delta function initial condition has a classical solution which, given the parameters in Eqs.S.34-S.35, takes the form

$$SNR(x, t) = \sqrt{\frac{N}{4\pi D(x)t}}\exp\Big[\frac{-\Big(\frac{n}{\bar{q}\ln q^{-1}}(q^{-x} - 1) - t\Big)^2}{4D(x)t}\Big], \tag{S.36}$$

where we have written directly $SNR(x, t) = S(x, t)/N^{1/2}$ and $D(x) = \bar{q}^{-1}q^{-x}/2$. Curiously, taking $D(x) = \bar{q}^{-1}q^{-x}/4$ provides a much better fit to the Markov simulations. Direct numerical simulation of the PDE's EqsS.23-S.24 reveals that the PDE itself fits the Markov simulations well and that Eq.S.36 with $D(x) = \bar{q}^{-1}q^{-x}/4$ is a very good approximate solution to the PDE (not shown). Therefore, in the analysis which follows, we will make use of Eq.S.36 with $D(x) = \bar{q}^{-1}q^{-x}/4$.

4.2 Readout of the memory signal: the role of correlations

We have already discussed the size of correlations in any one memory stage. Specifically, if the total number of synapses is N in a system with n stages, then the variance of the signal in stage i is $(N/n)(1 - p_i^2(t))$ which we have approximated as N/n. When reading out the memory signal from multiple stages we must be concerned with correlations in the fluctuations between different stages; these correlations will increase the variance of the signal.

We expect positive noise correlations in the signal between stages since the state of a synapse in stage i depends on the state of a synapse in stage $i-1$ whether it correctly encodes the signal or not. We can estimate this correlation by noting that the probability that both synapses are in the same state (above and beyond the chance probability of 1/4) at a time t is $q_i(1-q_{i-1})$. This is so because the synapse in stage i must have switched state to match the state of the synapse in stage $i-1$, and the synapse in stage $i-1$ cannot yet have switched state. Similar reasoning tells us that the correlation between stages i and $i-2$ is proportional to $q_i q_{i-1}(1-q_{i-1})(1-q_{i-2})^2$. The complete correlation matrix depends on such correlations of all orders.

In any case, we can write, for the total variance of the readout signal

$$\begin{aligned} \sigma^2 &= \sum_{i=1}^{n} \sigma_i^2 + \sum_{i=1}^{n} \sum_{j \neq 1} \sigma_{ij}^2, \\ &= \sum_{i=1}^{n} \frac{N}{n} + \sum_{i=1}^{n} \sum_{j \neq i} \sigma_{ij}^2, \\ &= N + \sum_{i=1}^{n} \sum_{j \neq 1} \sigma_{ij}^2. \end{aligned} \tag{S.37}$$

If the covariances σ_{ij}^2 are of the same order as the variances σ_i^2 then the total variance will be dominated by these covariances since there are $n(n-1)$ of them. However, these covariances are proportional to the learning rates, as we explain above, and so are not of order one. How can we estimate them? It is not hard to estimate the covariance between adjacent stages using the arguments above. It can be caluclated explicitly to give $\sigma_{i,i-1} = q_i(1-q_{i-1})\frac{N}{n}$. However, how do we take into account the contribution from all other stages? A simple calculation can give us a hint. The correlation between stage 1 and stage i is proportional to $q_1 q_2 \ldots q_i$. In our model we use $q_i = q^{(i-1)/(n-1)}$ which means that the correlation is proportional to $1 \cdot q^{1/(n-1)} \cdot q^{2/(n-1)} \cdot \ldots \cdot q^{(i-1)/(n-1)} = q^{\frac{1}{n-1}\sum_{j=1}^{i-1} i} = q^{\frac{i^2-i}{2(n-1)}}$. The idea is to sum this quantity over all i to see how the total cross-correlation scales as a function of n. This can be done by taking i as a continuous variable x and integrating from 1 to n, which gives the integral $\int_1^n dx e^{-\alpha(x^2-x)/n}$, where α is a constant. If we assume n is large and take $y = (x-1)\sqrt{\alpha/n}$ we can write this integral as $\sqrt{n/\alpha}\int_0^\infty dy e^{-y^2}$. This shows that adding up the small pairwise correlations between stages over all stages give a total contribution

of the order $\sqrt{n}$. Therefore, we can try approximating the total covariance as the covariance between adjacent layers, magnified by a factor $\sqrt{n}$. This gives

$$\begin{aligned}
\sigma^2 &= N\Big(1 + \frac{2}{\sqrt{n}}\sum_{i=1}^{n-1} q_{i+1}(1-q_i)\Big), \\
&= N\Big(1 + \frac{2}{\sqrt{n}}\sum_{i=1}^{n-1} q^{i/(n-1)}(1-q^{(i-1)/(n-1)})\Big). \qquad \text{(S.38)}
\end{aligned}$$

where the factor 2 is because $\sigma_{ij}^2 = \sigma_{ji}^2$.

Fig.S.6 shows that Eq.S.38 describes the true covariance quite well. In Fig.S.6 we show the results of numerical simulations of the stochastic memory transfer model for different values of q, where the learning rates are $q_i = q^{(i-1)/(n-1)}$ and there are 1000 synapses per stage. We track a memory which is never presented and hence the signal here has mean zero. To generate one data point we do 10 simulations of 1000 time steps each. For each simulation we calculate the noise (standard deviation) and then calculate the mean and standard deviation of this noise over the 10 simulations. We then plot the mean standard deviation normalized by the expected standard deviation if the noise were uncorrelated, which is just $\sqrt{N}$, the square root of the total number of synapses. Error bars show one standard deviation in the measurement of this normalized noise level over the ten trials. The solid lines are from Eq.S.38.

Given this good agreement between Eq.S.38 and numerical simulation of the stochastic model, we now study Eq.S.38 further. Specifically, we will consider two cases which correspond to the two types of readout we will consider in subsequent sections: 1 - a naive readout of all stages and 2 - a readout of only a subset of stages.

4.2.1 Correlations in a naive readout

In this case, doing the sum in Eq.S.38 explicitly leads to the following equation

$$\sigma^2 = N\Big(1 + \frac{2q^{1/(n-1)}}{\sqrt{n}}\Big[\frac{(1-q)}{(1-q^{1/(n-1)})} - \frac{(1-q^2)}{(1-q^{2/(n-1)})}\Big]\Big) \qquad \text{(S.39)}$$

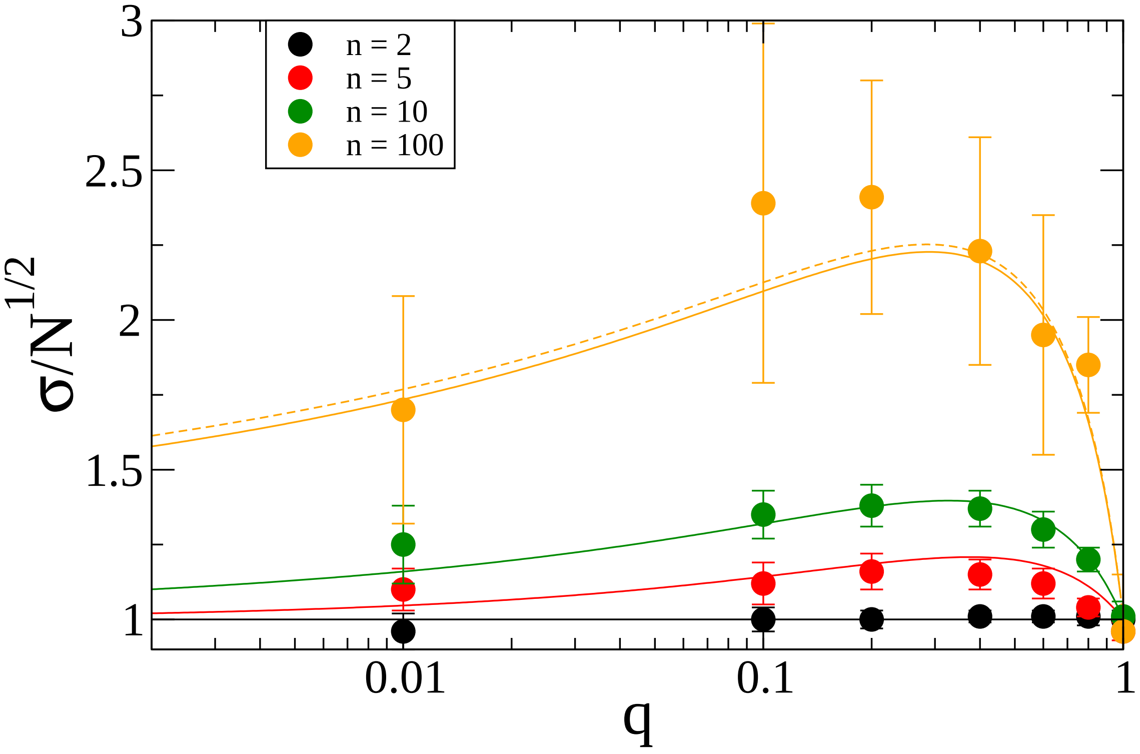

Fig. S.6: The noise level in the memory transfer model as a function of the slowest learning rate. The symbols are the means of 10 simulations of 10^3 time steps and error bars indicate the standard deviation. The curves are the analytical approximation. The learning rate in stage i is $q_i = q^{(i-1)/(n-1)}$. Each stage has 1000 synapses.

If we assume that $\ln(q^{-1})/n \ll 1$, i.e. the scaling used in the continuous space approximation, then this equation gives, to leading order

$$\sigma^2 = N\Big(1 + \frac{(1-q)^2}{\ln(q^{-1})}\sqrt{n}\Big). \tag{S.40}$$

Eq.S.40 is used to make the dashed curve in Fig.S.6. To put some numbers to this expression, taking $n = 10$ and $q = 10^{-2}$ gives $\sigma = 1.3\sqrt{N}$, while $n = 100$ and $q = 10^{-4}$ gives $\sigma = 1.4\sqrt{N}$. In any case, the noise clearly increases as a function of the number of stages.

4.2.2 Correlations in a readout of only a subset of stages

If we only readout from stage k to stage $k + w$, then Eq.S.38 gives, for $\ln(q^{-1})/n \ll 1$

$$\sigma^2 = N\Big(1 + \frac{2w}{\sqrt{n}} q^{k/n}(1 - q^{k/n})\Big). \tag{S.41}$$

Eq.S.53 tells us that the size of the correlation depends on the width of the readout. Furthermore, when k is close to one or n, then the correlations vanish. We will make use of this formula in Section 4.4 *Optimal readout of the memory signal.*

4.3 Naive readout of the memory signal

Naively one can simply readout the signal of all of the synapses. This is equivalent to integrating $SNR(x,t)$ over x. In this case one can perform the integral analytically in the limit $\epsilon = \ln q_s^{-1}/n \ll 1$. One obtains

$$SNR(t) = \frac{\bar{q}N^{1/2}}{\sqrt{(1 + \frac{(1-q)^2}{\ln(q^{-1})}\sqrt{n})2(n + \bar{q}\ln(1/q_s)t)}}\Big(1+\mathrm{erf}(\frac{\frac{n}{\bar{q}\ln(1/q_s)}(q_s^{-1} - 1) - t}{\sqrt{q_s^{-1}t/\bar{q}}})\Big), \tag{S.42}$$

which also makes use of Eq.S.40.

4.4 Optimal readout of the memory signal

As for the heterogeneous model we can maximize the SNR by only reading out a fraction of stages. In the case of the consolidation model, it turns out that the optimal readout is to follow the memory trace as it propagates and

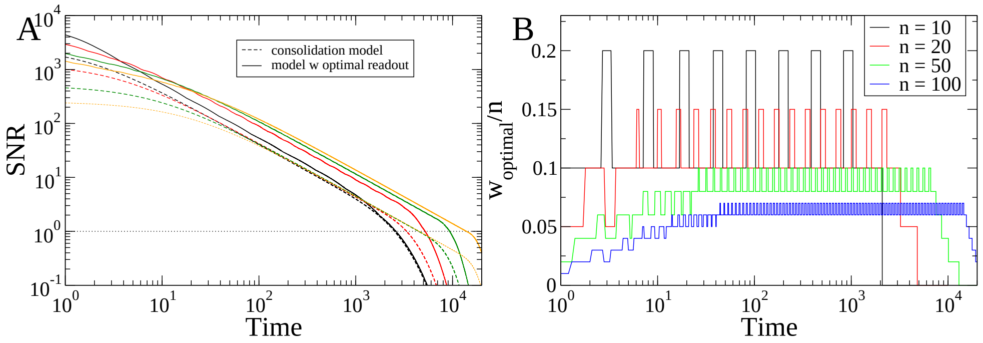

Fig. S.7: A. The SNR for the consolidation model reading out all ensembles (dashed lines) as well as using an optimal readout (solid lines). The total number of synapses is $N = 10^9$ which are divided into $n = 10, 20, 50$ and 100 ensembles (black, read, green, blue). Here $q_i = \bar{q}q^{(i-1)/(n-1)}$ with $\bar{q} = 0.8$ and $q = 0.001$. B. The width of the optimal readout for different values of n. Note that for fixed n that width reaches a constant value at early times, i.e. it is independent of time for long times (until the pulse exits the system).

read out the stages near the maximum of the pulse. The fraction of stages to be read out as a function of the total system size decreases for increasing n as shown in Fig.S.7.

We can therefore calculate the SNR of the optimal readout by assuming that the bounds of the integral move along with the pulse. This also allows one to calculate the optimal width. For any bounds $a(t)$ and $b(t)$ which vary in time, the SNR is just

$$SNR(t) = \frac{1}{\sqrt{b(t) - a(t)}} \int_{a(t)}^{b(t)} dx SNR(x,t) \tag{S.43}$$

We then choose the bounds in order to track the pulse. We take $a(t) = \ln(\bar{q}^{-1}(1+\epsilon t))/\ln q^{-1} - \mu$ and $b(t) = \ln(\bar{q}^{-1}(1+\epsilon t))/\ln q^{-1} + \mu$ and then perform a change of variables by defining $z = x - \ln \bar{q}^{-1}(1+\epsilon t)/\ln q^{-1}$, where $\epsilon = \ln q^{-1}/n$. This converts the integral to the following form

$$SNR(t) = \sqrt{\frac{N}{2\pi\mu t}} \int_{-\mu}^{\mu} dz \frac{q^{z/2}}{\sqrt{t(1+\epsilon t)}} \exp\Big[- \frac{((q^{-z}(1+\epsilon t) - 1)/\epsilon - t)^2}{q^{-z}(1+\epsilon t)t} \Big]. \tag{S.44}$$

Now one takes a large time limit which simplifies things considerably. In fact, it completely eliminates the time dependence of the integral, which

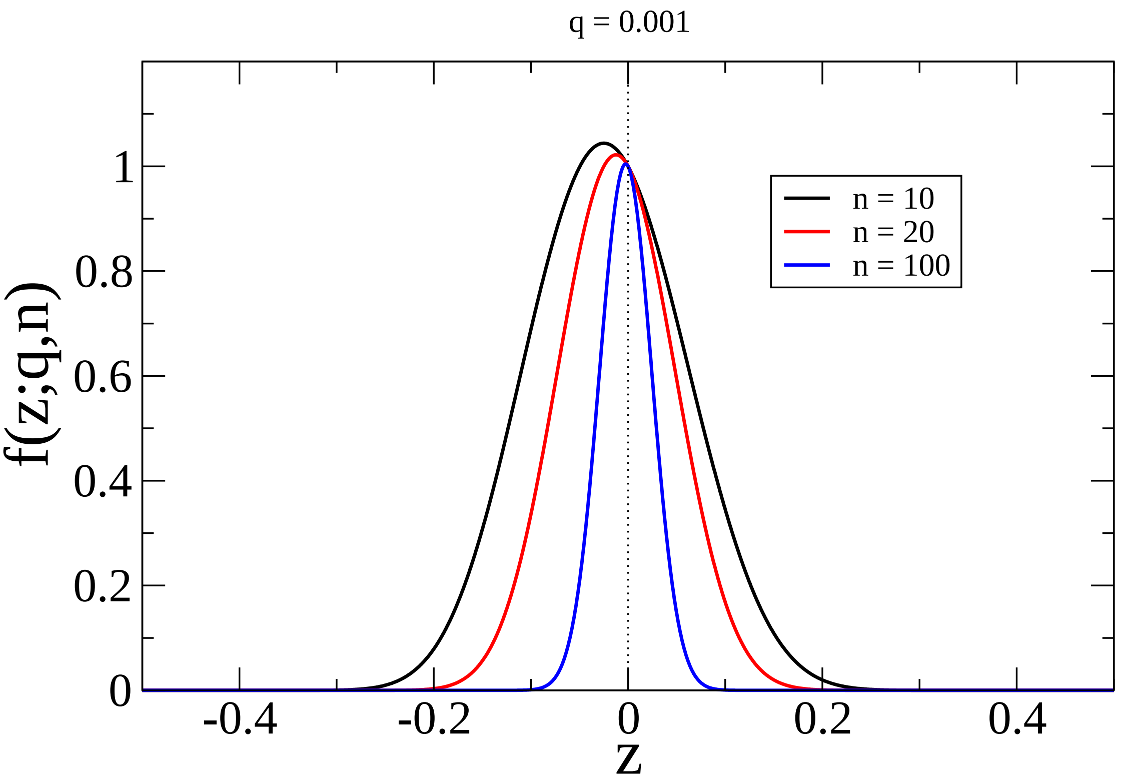

Fig. S.8: The integrand f(z) from the integral in Eq.S.45

shows that the optimal width is independent of time at long times, as can be observed numerically in Fig.S.7B. Doing this yields the formula

$$SNR(t) = \frac{\sqrt{N}}{\sqrt{\pi\epsilon}t}\int_{-\mu}^{\mu} dz q^{z/2} \exp\Big[-\frac{(q^{-z}+q^{z}-2)}{\epsilon}\Big]. \qquad \text{(S.45)}$$

The integrand is not a symmetric function so one wouldn't be justified in integrating from $-\mu$ to μ in general. However, for small ϵ it is very close to symmetric. Fig.S.8 shows what this function looks like for several values of n. The position of the maximum is to the left of zero, but it turns out that this position scales like ϵ while the limits of integration μ will end up scaling like $\sqrt{\epsilon}$ so it is a higher order effect.

One can find the optimal μ by taking the derivative of the SNR with

respect to μ and setting it to zero. Taking the integrand to be $f(z)$ this gives

$$\begin{aligned}\frac{\partial SNR}{\partial \mu} &= -\frac{\sqrt{N}}{2}\frac{1}{\sqrt{2\pi\epsilon}\mu^{3/2}t}\int_{-\mu}^{\mu} dz f(z) \\ &\quad +\frac{\sqrt{N}}{\sqrt{2\pi\epsilon\mu}t}\Big(f(\mu)+f(-\mu)\Big)=0,\end{aligned} \tag{S.46}$$

This can be rewritten as

$$\frac{1}{\mu}\int_{-\mu}^{\mu} dz f(z) = 2\Big(f(\mu)+f(-\mu)\Big). \tag{S.47}$$

Now we assume that μ can be expanded in a series in ϵ. The idea is that as the system size grows, the optimal width will decrease as a fraction of the total system size, which is what is observed numerically, see FigS.7B. We have already normalized x by dividing by n, so to compare with the numerically determined optimal width we will have to multiply by n. We are only interested in the first term of the series so we take

$$\mu = \epsilon^{\alpha}\mu_0, \tag{S.48}$$

and we will need to solve for the scaling α and the value μ_0. The right hand side is easier. We expand $f(\mu)$

$$\begin{aligned}f(\mu) &= q^{\mu/2}e^{-(q^{-\mu}+q^{\mu}-2)/\epsilon}, \\ &= e^{-\epsilon^{2\alpha-1}\mu_0^2(\ln q^{-1})^2} + h.o.t,\end{aligned} \tag{S.49}$$

where $h.o.t$ means higher order terms. The left hand side is trickier only in the sense that we should do a change of variables $y = z/\mu$ to put the small parameter in the integrand. Then we follow much as above. Finally, we end up with

$$\frac{\pi}{\epsilon^{(2\alpha-1)/\alpha}\mu_0 \ln q^{-1}}\text{erf}\Big(\epsilon^{(2\alpha-1)/2}\mu_0 \ln q^{-1}\Big) + h.o.t = 4e^{-\epsilon^{2\alpha-1}\mu_0^2(\ln q^{-1})^2} + h.o.t \tag{S.50}$$

We still do not know the proper scaling since α is an unknown. However, we can make a guess and see what happens. If we choose $\alpha < 1/2$ then we get something on the left which scales like $\epsilon^{1/2-\alpha}$ but on the right we get something exponentially small, so they can not possibly balance. If we take $\alpha > 1/2$ we get something on the left which is large and goes like $\epsilon^{1/2-\alpha}$

again, but on the right we have something which to leading order is just 4, so that also cannot be balanced. Therefore it must be that $\alpha = 1/2$. With that choice the equation simplifies to

$$\mathrm{erf}(x) = \frac{4}{\sqrt{\pi}} x e^{-x^2}, \tag{S.51}$$

where $\mu_0 = x/\ln q^{-1}$. Amazingly, the solution x is nearly equal to 1 (it differs from 1 by about 1%). Therefore, taking $x = 1$, the optimal width is

$$\begin{aligned} w &= 2\mu, \\ &= \sqrt{\epsilon}\mu_0, \\ &= \frac{2}{\sqrt{n \ln q^{-1}}}. \end{aligned} \tag{S.52}$$

as a fraction of the system size. In terms of the number of stages it is $2\sqrt{n/\ln q^{-1}}$.

Now, in order to evaluate the SNR we must determine the size of the correlations in the noise, given by Eq.S.53. Using the equation for the width of the optimal readout Eq.S.52, we find that the total variance is

$$\sigma^2 = N\Big(1 + \frac{4}{\sqrt{\ln (q^{-1})}} q^{k/n}(1 - q^{k/n})\Big), \tag{S.53}$$

which is independent of the number of stages n. Furthermore, Eq.S.53 reaches a maximum of $\sigma^2_{\max} = N\Big(1+\frac{1}{\sqrt{\ln (q^{-1})}}\Big)$ at stage $k = n\frac{\ln 2}{\ln (q^{-1})}$. This means, for example, that given the parameters in Fig.3 of the main text ($q = 0.0001$), the noise level reaches a maximum of $1.15\sqrt{N}$ when the signal is near stage 7, i.e. at very early times. Fig.S.10 shows numerical confirmation of this. It shows the SNR using the optimal readout for the same parameters as in Fig.3 of the main text (number of stages n=100) both assuming there are no correlations (solid line) and including the effect of correlations (dashed line). The correlations are clearly very small for the optimal readout. Specifically, they do not affect the scaling of the intitial SNR or the memory lifetime, and only very weakly the point at which the SNR crosses that of the heterogeneous model. Therefore, for simplicity, we will approximate the noise level simply as $\sqrt{N}$.

Now we can evaluate the SNR by plugging the formula for μ into Eq.S.45. Keeping only the first order term in ϵ gives

$$SNR(t) = \frac{N^{1/2} n^{1/4}}{\sqrt{2}(\ln q^{-1})^{3/4} t} \mathrm{erf}(1). \tag{S.54}$$

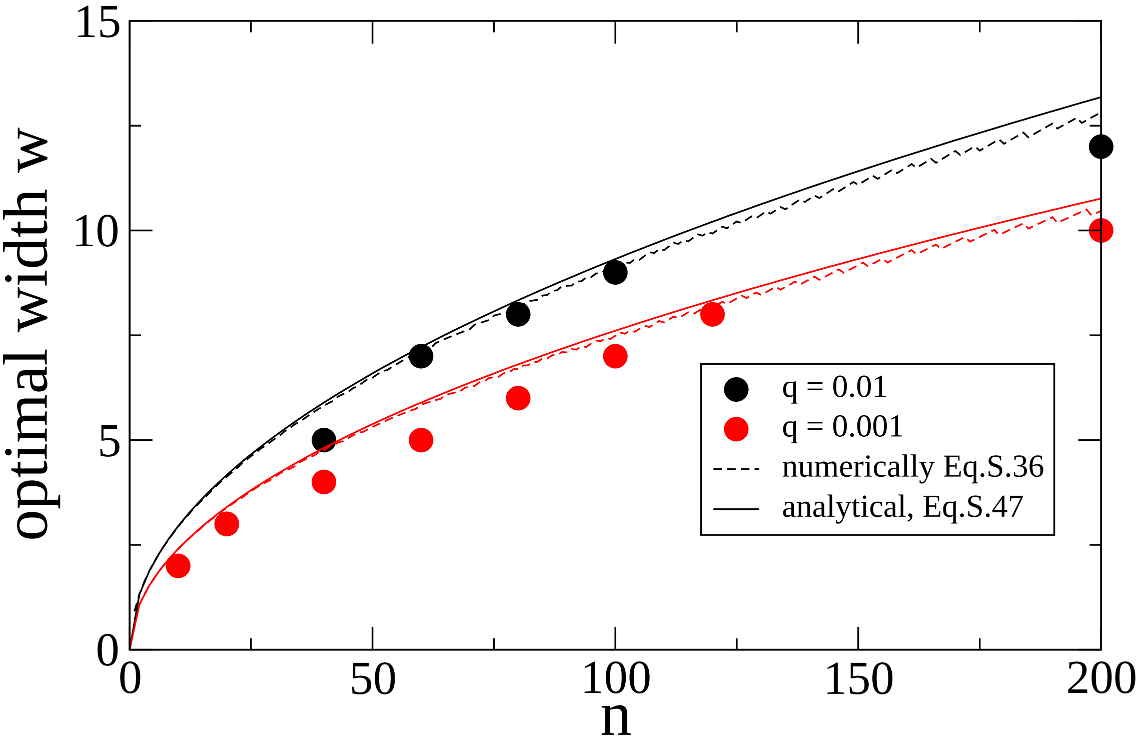

Fig. S.9: The optimal width from simulations (symbols), from evaluating the integral Eq.S.45 and from the formula Eq.S.52.

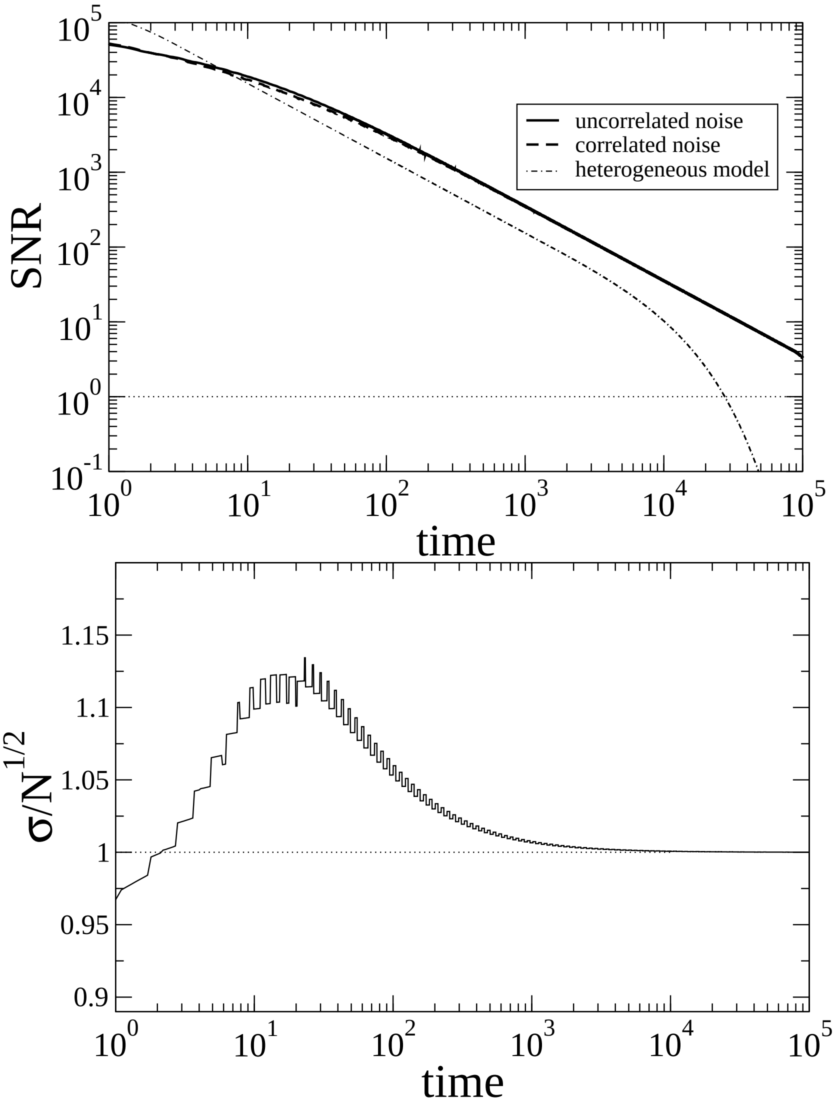

Fig. S.10: Top: The SNR using the optimal readout, ignoring correlations (solid line) and including the effect of correlations (dashed line). The SNR of the heterogeneous model is also shown (dash-dot line). Bottom: The total normalized noise strength as a function of time. All parameters are the same as in Fig.3 of the main text, with $n = 100$.

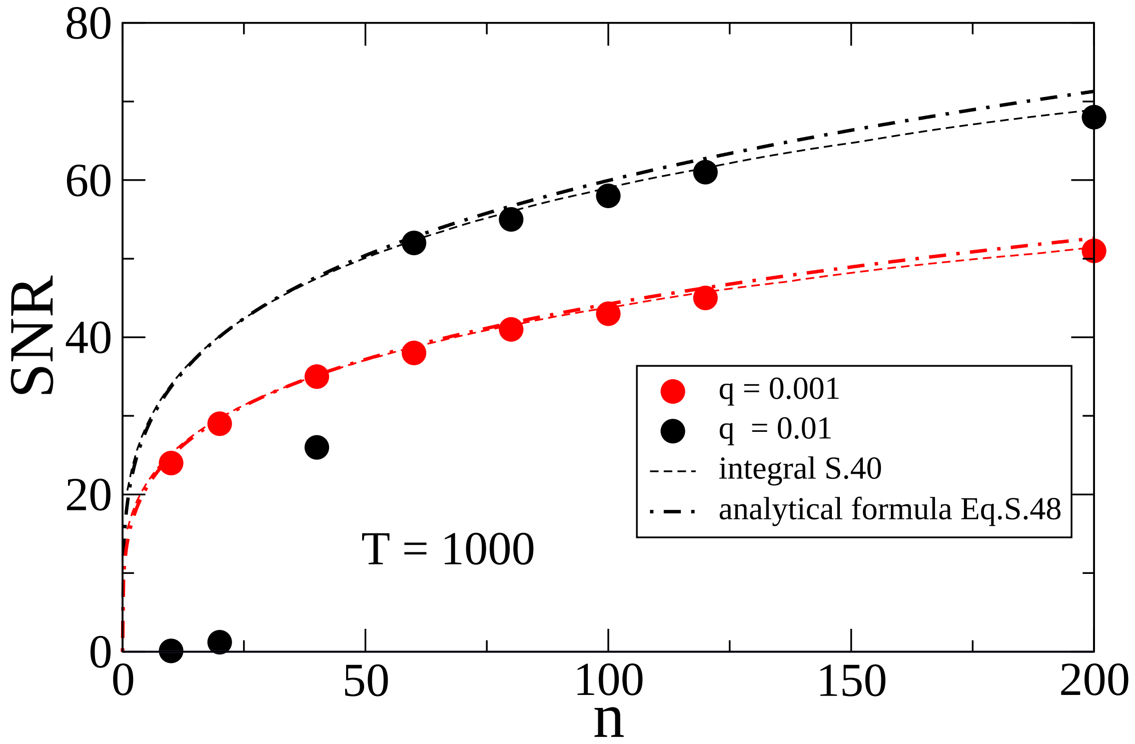

Fig. S.11: The SNR for two values of q at time T=1000. Numerics are symbols, dashed lines are from the integral Eq.S.45 and the dot-dashed lines are from Eq.S.54.

This means that the decay of the SNR given the optimal readout is also a power law with power equal to one. However, unlike the naive readout, where at long times the SNR was independent of n, here the SNR actually increases as $n^{1/4}$. Eq.S.54 fits the numerical results very well, see Fig.S.11

4.5 A plausible read-out which is nearly optimal

The optimal readout we have implemented is computationally trivial to calculate, but it is unclear how it might be carried out in the brain. Here we show that it can, in fact, be implemented approximately through a simple rule. Specifically, for each stage there is a threshold value of the SNR below which the signal from that stage is no longer read out. We assume that this threshold can be learnt and is then held fixed for all memories.

In order to make the readout nearly optimal, we choose a threshold for stage k, which has a position $x = x_k$ equal to the value of $SNR(x_k, T_k)$ where T_k is the time at which, using the optimal readout, we just cease to read out the signal in stage k. This time is given by the lower bound $a(t)$ in the integral Eq.S.43. That means T_k is defined implicitly through the relation $a(T_k) = x_k$. This can be solved for T_k, yielding

$$T_k = (q^{-(x_k+\mu)} - 1)/\epsilon, \tag{S.55}$$

where μ is the optimal one we already calculated. Plugging this into the formula for $SNR(x_k, t)$ gives the threshold only as a function of x_k

$$SNR(x_k) = \sqrt{\frac{N\epsilon}{\pi q^{-x_k}(q^{-(x_k+\mu)} - 1)}} \exp\left(-\frac{\left((q^{-x_k} - 1)/\epsilon - (q^{-(x_k+\mu)} - 1)/\epsilon\right)^2}{q^{-x_k}(q^{-(x_k+\mu)} - 1)/\epsilon}\right). \tag{S.56}$$

This formula can be simplified for x_k not too close to 0 which is equivalent to assuming long times. Then $q^{-x_k} - 1 \sim q^{-x_k}$. Also we plug in the optimal $\mu = \sqrt{\epsilon}/\ln q^{-1}$ and then expand in the small parameter ϵ. The leading order term is

$$SNR(x_k) = \sqrt{\frac{N \ln q^{-1}}{\pi n}} \frac{q^{x_k}}{e}, \tag{S.57}$$

which is exponentially decreasing in x_k.

Using this readout with optimally adjusted fixed thresholds would be exactly equivalent to the optimal readout if the integrand $f(z)$ were symmetric. Since it's very nearly symmetric for small ϵ at long times the readout is essentially optimal. We will therefore use the optimal readout in what follows.

4.6 The performance of the memory transfer model: Crossing times, SNR and memory lifetimes (Fig.3)

The curves of SNR versus time in the main panel of Fig.3 in the main text are generated via the meanfield model (the ODEs in continuous time, discrete stages), for both the heterogeneous system (dashed lines) and the consolidation model (solid lines). They represent the curves obtained using the optimal readout described in detail in previous sections. Three measures of interest are indicated by colored circles in the main panel of Fig.3: 1 - the

time at which the SNR of the consolidation model crosses (and thereafter is larger than) the SNR in an equivalent heterogeneous model, denoted T_c for *crossing time*, 2 - The SNR of the consolidation model at long-times in the powerlaw regime, and 3 - The lifetime of a memory, i.e. the time at which the SNR of a memory dips below 1, denoted T_{LT}. Here we describe how these measures are calculated.

4.6.1 The crossing time T_c

The SNR for the heterogeneous model and the consolidation model with identical $\bar{q}$, q, N, and n are calculated numerically from the meanfield model, using the optimal readout. The time at which the SNR of the consolidation model crosses and exceeds that of the heterogeneous model is called T_c. This is plotted in the inset of Fig.3 of the main text versus n. As shown in the section *The continuous-time approximation*, for $n = 2$ one can derive the crossing-time analytically to find $T_c \sim 1/\bar{q}$ as long as the learning rates are similar. For $n > 2$ it is no longer possible to calculate T_c analytically as the SNR of many stages contribute to the total SNR in a nontrivial fashion. Nonetheless, numerically, it appears that T_c always occurs at very early times compared to the memory lifetimes for all parameter values we have explored.

4.6.2 The SNR in the powerlaw regime

The SNR shown in the lower lefthand panel of Fig.3 in the main text as a function of n for different slowest learning rates q, is calculated numerically from the meanfield equations (symbols) and given by the analytical formula Eq.S.54 (lines). The SNR is evaluated at different times for the three different learning rates. The upshot is that the SNR in the powerlaw regime is proportional to $N^{1/2}$ and $n^{1/4}$.

4.6.3 Memory lifetime

The memory lifetime can be calculated analytically from Eqs.S.29 and S.54. Specifically, the powerlaw regime ends once the pulse reaches the last stage. This occurs at a time T which is given by Eq.S.29. The SNR at this point is then given by Eq.S.54 evaluated at time T, as long as the SNR is greater than one. The memory trace then decays exponentially with a learning rate equal to that of the last stage. Finally, the SNR reaches a value of one at a

time $T_{LT} = T + T_{exp}$ which can be written

$$T_{LT} = \frac{n}{\bar{q}q \ln q^{-1}} + \frac{1}{\bar{q}q} \ln \left[\frac{N^{1/2}}{2^{1/2} n^{3/4}} \bar{q}q \cdot \mathrm{erf}(1) (\ln q^{-1})^{1/4} \right]. \quad \text{(S.58)}$$

If the SNR drops below one already in the powerlaw regime, then the lifetime can be calculated from Eq.S.54 alone and is

$$T_{LT} = \frac{N^{1/2} n^{1/4}}{2^{1/2} (\ln q^{-1})^{3/4}} \mathrm{erf}(1). \quad \text{(S.59)}$$

If the SNR drops below one even before the powerlaw regime is reached then Eq.S.59 is no longer valid. Eqs.S.58 and S.59 are used to generate the curves in the lower right hand panel of Fig.3.

To reiterate, if the initial number of synapses is large, for small n we expect that the final decay of the last stage will occur before the SNR reaches 1. Therefore Eq.S.58 is valid. As n increases the curve will move downwards such that the SNR reaches 1 already in the powerlaw regime and Eq.S.59 is valid. Finally, as n increases further, at some point the SNR drops below 1 even before the powerlaw regime. This means that in this limit fewer and fewer stages have a SNR greater than 1. In fact, in the limit of $n \to \infty$, the SNR of the first stage will already be less then 1 at time $t = 0$. At this point the lifetime will also be zero. Therefore, the lifetime must decrease with increasing n outside of the powerlaw regime. These trends can clearly be seen in Figs.S.12 and S.13 which show the lifetime as a function of the number of stages n for different learning rates q and different numbers of synapses N. In particular, note how the green curve in Fig.S.13 reaches a maximum and then decreases for large n.

Note that a scaling of the memory lifetime as $T_{LT} \sim nN^{1/2}$ can be achieved by taking $q \propto 1/N^{1/2}$, and ensuring that the SNR does not drop below one in the powerlaw regime, i.e. Eq.S.58 is valid. This is illustrated in Figs.S.12 and S.13 for three different values of q which scale as $q \propto 1/N^{1/2}$. For each value of q three different numbers of synapses are considered: $N = 10^5$, 10^7 and 10^9 (green, red and black respectively). For each case the memory lifetime was determined numerically for the consolidation model (solid circles) and the heterogeneous model (open squares), both using optimal readout. The lines are the predictions for the consolidation model, Eqs.S.58-S.59. As described above there are several qualitatively distinct scaling regimes of the lifetime as a function of the number of stages. For

small n the decay of the slowest stage is of the same order or larger than the time it takes for the pulse to travel to reach the last stage, i.e. both terms in Eq.S.58 are of the same order. This can even lead to a minimum in the lifetime as a function of n which would indicate a non-optimal number of stages. For larger n the lifetime is dominated by the propagation time of the pulse and ends, in fact, only when the pulse leaves the system through the slowest stage. In this regime the scaling of the memory lifetime is approximately linear in n. For $n > n_{max}$, the SNR drops below one even before the pulse leaves the system and lifetimes are given by Eq.S.59, i.e. the lifetime scales as $n^{1/4}$. Finally, as n increases further, the SNR drops below one already before the powerlaw regime and the lifetimes decrease again.

While it is clear from Fig.S.12 that the consolidation model outperforms the heterogeneous model without interactions for all n, the greatest improvement is achieved in the intermediate regime in which lifetimes scale as n. As described in the previous paragraph, this regime is bounded below by n_{min}. For $n < n_{min}$ the propagation of the pulse to the last stage occurs too quickly compared to the slowest time scale. This only occurs for relatively small numbers of stages. The upper bound on the linear regime n_{max} is given by setting the lifetimes given by Eq.S.58 and Eq.S.59 equal. Doing this and taking the limit $N \to \infty$ yields the scaling of maximum number of stages with the number of synapses as $n_{max} \propto \sqrt{\ln N}$. Fig.S.14 shows n_{max} as a function of the square root of the log of the number of synapses N, from Eqs.S.58-S.59, confirming the scaling for large enough N. Shown is the case of $q = 30/N^{1/2}$ for which $n_{min} \sim 5$. Therefore, there is a large range in n for which lifetimes scale as $nN^{1/2}$. Note that n_{max} scales only weakly with N, such that if the number of synapses is increased from 10^4, which would be the case for ~ 300 neurons with ten percent sparseness, to 10^{14}, which is approximately the total number of synapses in an adult human brain, the number of stages is only doubled.

4.7 Comparison between the multi-stage memory transfer model and homogenous (single stage) models

For a fair comparison of the memory capacity of multi-stage and single stage homogeneous models it is important to take into account that the amount of information stored per memory is different in the two models. Indeed, in the multi-stage model the information is stored only in the first stage, which

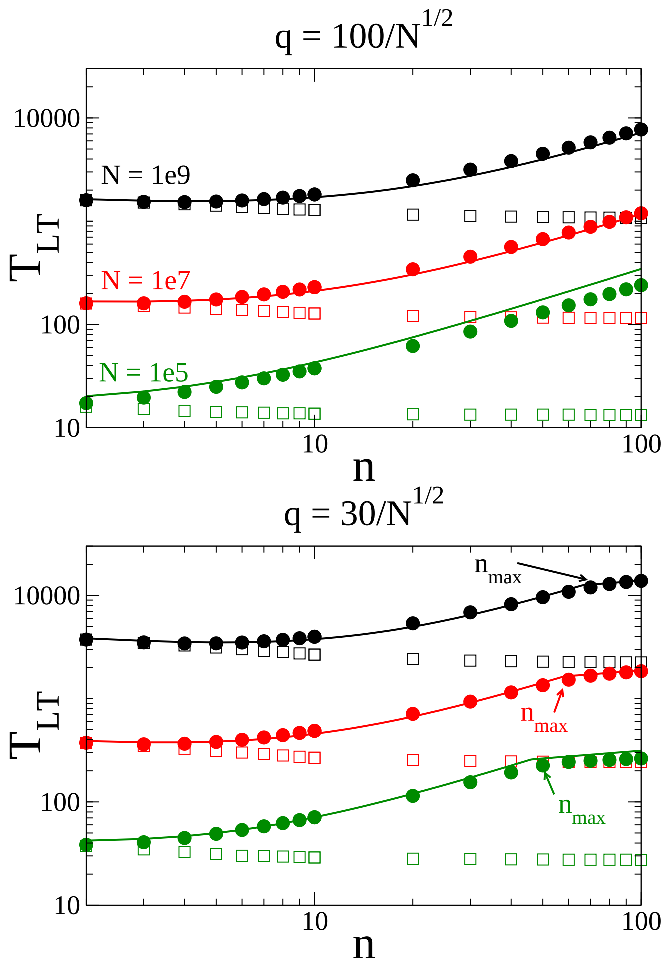

Fig. S.12: Memory lifetimes as a function of the number of stages n for different learning rates ($\bar{q} = 0.8$, $q = 100/N^{1/2}$, $30/N^{1/2}$ top to bottom), and different numbers of synapses ($N = 10^5$, 10^7 and 10^9, green, red and black respectively). Symbols are from simulation of the meanfield model (solid circles and open squares are consolidation model and heterogeneous model respectively). Lines are Eqs.S.58-S.59. The maximum number of stages for the linear regime n_{max} is shown in the bottom panel.

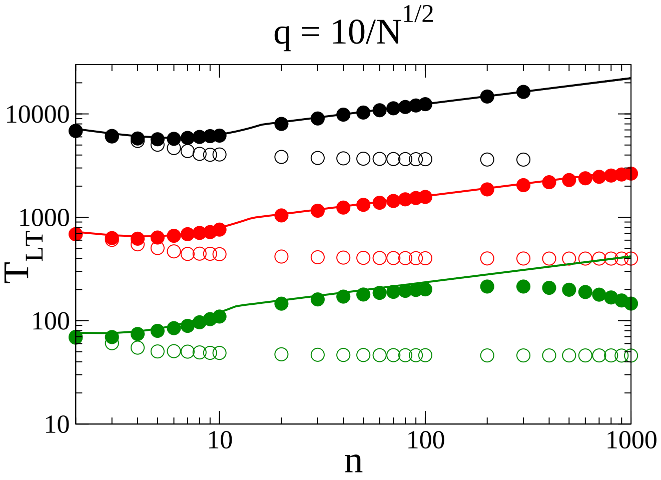

Fig. S.13: Memory lifetimes as a function of the number of stages n for different learning rates ($\bar{q} = 0.8$, $q = 10/N^{1/2}$), and different numbers of synapses ($N = 10^5$, 10^7 and 10^9, green, red and black respectively). Symbols are from simulation of the meanfield model (solid circles and open squares are consolidation model and heterogeneous model respectively). Lines are Eqs.S.58-S.59.

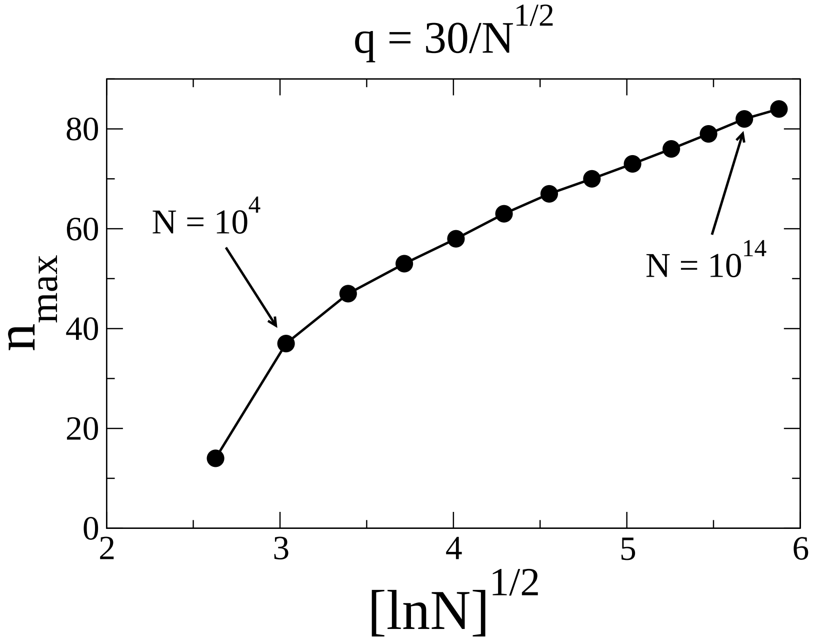

Fig. S.14: The maximum number of stages in the linear regime as a function of $\sqrt{\ln N}$ is a straight line for large enough N.

contains N/n synapses (n is the number of stages), whereas in the single stage model all N synapses are available to store new memories. As we assumed that the memories are random and uncorrelated and that potentiating events are equally probable as depressing events, then each synapse can store up to one bit of information per memory. Hence, the first stage of the multi-stage model can store up to N/n bits of information, which can be significantly less what is potentially storable in a single stage model.

In order to compare the single stage and the multistage model we need to consider a situation in which the amount of information stored per memory is the same. In order to do so, consider a single stage model in which the patterns of synaptic modifications are sparse. More specifically, each synapse is modified with a probability q_f. In this case the amount of information contained in the pattern of synaptic modifications is:

$$I_{sg} = N[-q_f \log_2 q_f - (1 - q_f) \log_2(1 - q_f)]$$

which, in the limit for $q_f \to 0$ can be approximated by $-Nq_f \log_2 q_f$. The information stored in each pattern of synaptic modifications is $I_{mg} = N/n$ bits in the case of the multistage model. For a fair comparison, we should choose q_f such that $I_{mg} = I_{sg}$. This means that $q_f \sim 1/n$.

As q_f decreases, the memory lifetime extends at the expense of the initial signal to noise ratio. Specifically:

$$SNR(t) \simeq \sqrt{N} q_f q e^{-q_f q t}$$

As it is clear from this formula, making the patterns of synaptic modifications sparser is equivalent to rescale the learning rate:

$$SNR(t) \simeq \sqrt{N} \tilde{q} e^{-\tilde{q} t}$$

where $\tilde{q} = q_f q$. The longest memory lifetime can be achieved for $\tilde{q} = 1/\sqrt{N}$, which produces an initial SNR that is order 1 (i.e. it does not scale with the number of synapses N).

The only difference with the single stage model in which we do not introduce any correction for equalizing the amount of information per memory, is that now $\tilde{q}$ can vary in a wider range, as it can go from 1 (fastest) to $q_f q_n$, which is approximately q_n/n. This means that the memory lifetime can in principle be as large as in the multistage model. However, it should be noted that any reduction of the learning rate leads to a decrease of an already small

initial signal to noise ratio. As shown in Figure S.15, even if one considers single stage models with timescales that are longer than q_n, all the SNR curves for single stage models are below the SNR of the multistage model. This is true for all times. This clearly indicates that there is a significant memory capacity advantage in using a more complex multistage model.

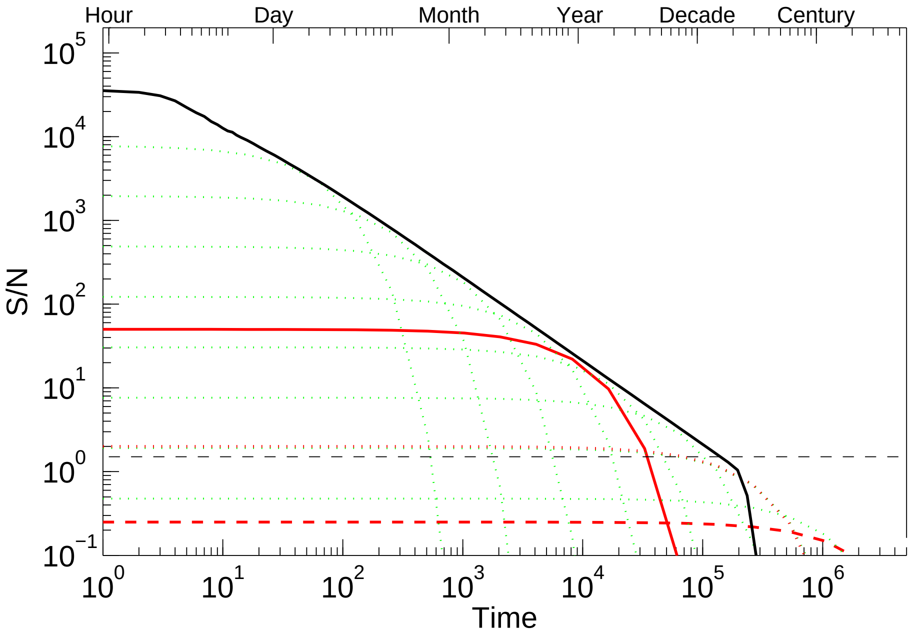

Fig. S.15: SNR of the multistage model (black) compared to the SNR of single stage models. Red solid line: SNR of a single stage model with the longest timescale of the multistage model (q_n) in the case in which the amount of information per memory is approximately n times larger than in the multistage model. All the other curves refer to single stage models in which the amount of information per memory is the same as in the multistage model. Dashed red line: single stage model with $q = q_n$. Dotted red line: single stage model with the same memory lifetime as the multistage model. Notice that the initial SNR is orders of magnitude smaller than in in the multistage model. Green dotted lines: SNR of single stage models with various learning rates. All SNR curves of single stage models are below the SNR of the multistage model for all times. Parameters: $n = 200$, $N = 10^{12}$.

5 The neuronal memory transfer model (Fig.3)

Here we provide a brief, qualitative description of the model. Details are given in the subsequent subsections. The neuronal model is once again a Markov model. The model consists of n stages of McCulloch-Pitts neurons. Neurons are recurrently connected within each stage, and connected in a feedforward fashion from one stage to the next. There are no feedback connections between stages. The model operates in two distinct modes of activity: 1 - encoding and 2 - transfer, see Fig.S.16 for an illustration. During encoding, a pattern of neuronal activity is imposed in stage 1 and the recurrent synapses are updated according to a simple Hebbian rule. During transfer, some fraction of neurons in each upstream stage is activated. This leads to activation in the downstream stage. An appropriate learning rule is implemented in order to update the recurrent synapses in the downstream stage such that they become more correlated with those in the upstream stage. That is, the upstream stage 'teaches' the downstream stage the correct synaptic weights.

5.1 The Markov model

There are n stages. Each stage is made up of N_{neuron} all-to-all coupled neurons. Each one of the $N = N_{\text{neuron}}^2 - N_{\text{neuron}}$ synapses (no self-coupling) can take on one of two non-zero values. Specifically, the synapse from neuron j to neuron i $J_{ij} \in \{J^+, J^-\}$, where $J^+ > J^-$. Furthermore, there are one-to-one connections from a neuron i in stage k to a neuron i in stage $k+1$. These connections are so strong that any presynaptic activity elicits postsynaptic activity without fail. In the initial condition, the synaptic matrices for all n stages are in the equilibrium state where any synapse is in the potentiated state J^+ or in the depressed state J^- with probability 1/2.

5.1.1 Encoding

A memory is encoded in stage 1. Specifically, one half of the neurons are randomly chosen to be activated ($s_i = 1$ if $i \in \{\text{active}\}$), while the remaining neurons are inactive ($s_i = 0$ if $i \in \{\text{inactive}\}$). A synapse J_{ij} is then potentiated to J^+ with a probability q_1 if $s_i = s_j$ and is depressed with probability q_1 if $s_i \neq s_j$. This encoding scheme is illustrated in Fig.S.16 for a simple model of two stages of four neurons each. After encoding a single memory, transfer

activity is simulated (see below) after which the next memory is encoded, and so on.

5.1.2 Transfer

A fraction f of neurons in stage 1 is activated at time t. Because of the powerful feedforward connections, the same subset of neurons is activated in stage 2. Specifically, a subset of fN_{neuron} neurons in stage 1 is stimulated at time t, i.e. $s_i^1(t) = 1$ for $i \in \{\text{active}\}$, while $s_i^1(t) = 0$ for the remaining neurons. At time $t+1$ then we have $s_i^2(t+1) = 1$ for the same subset as in stage 1. The recurrent connectivity may lead to postsynaptic activation in stage 1 neurons. Each neuron i receives an input $h_i^1(t) = \sum_{j=1}^{N} J_{ij}^1 s_j^1(t)$. If $h_i > \theta_i$, where θ_i is a threshold, then neuron i is activated at time $t+1$, i.e. $s_i^1(t+1) = 1$. Again, because of the powerful feedforward connections, the same subset of neurons in stage 2 is activated, i.e. $s_i^2(t+2) = 1$.

We take $\theta_i = \theta$ to be the same for all neurons and assume that it can take one of two values $\theta \in \{\theta_l, \theta_h\}$ with equal likelihood during each replay. These values correspond to a low threshold and a high threshold respectively. We assume that the recurrent connections in stage 2 have been 'dialed down' to the point where they do not influence the postsynaptic activation, i.e. the recurrent connections in stage 2 are unimportant for this mode of dynamics. This is the case if all of the recurrent synapses in stage 2 are multiplied by a modulatory factor α which is very small. We now need a plasticity rule for synapses in stage 2. If $\theta = \theta_l$, then $J_{ij}^2 = J^-$ at time $t+2$ with probability q_2 if and only if $s_j^2(t+1) = 1$ and $s_i^2(t+2) = 0$, otherwise the synapse is unchanged. This says that if, despite the low threshold, the presynaptic activity did not elicit postsynaptic activity, then the synapses in stage 1 must have been weak, therefore I will depress the corresponding synapses in stage 2. If $\theta = \theta_h$, then $J_{ij}^2 = J^+$ with probability q_2 if and only if $s_j^2(t+1) = 1$ and $s_i^2(t+2) = 1$. Otherwise the synapse is unchanged. This says that if, despite the high threshold, the presynaptic activity did elicit postsynaptic activity, then the synapses in stage 1 must have been strong, therefore I will potentiate the corresponding synapses in stage 2. At time $t+2$ all of the neurons in stage 1 are silenced, i.e. $s_i^1(t+2) = 0$ signalling the end of the current 'replay'. The interaction between stage 2 and stage 3 is analogous. That is, the factor α is set to 1 for stage 2 and dialed down for stage 3 and the synaptic weights are transferred according to the process described above. This is repeated until the synapses in the final stage are changed.

Then the entire replay process is repeated T times before a new memory is imprinted on stage 1. This process of transfer is illustrated in Fig.S.16 for the simpled case of two stages of four neurons each.

5.1.3 The fraction of synapses transferred during replay

The postsynaptic activation due to recurrent connections depends on the presynaptic input. This input depends on the number of neurons activated, which in this example will be exactly fN_{neuron}, and the state of the synapses which changes over time. The distribution of recurrent inputs is binomial, but for fN_{neuron} large enough is nearly Gaussian. The input due to potentiated synapses is $I^+ = \mu^+ + \sigma^+ c$ where c is a random variable with zero mean and unit variance, $\mu^+ = fN_{\text{neuron}}J^+/2$ and $\sigma^+ = J^+\sqrt{fN_{\text{neuron}}/4}$. The input due to depressed synapses is $I^- = \mu^- - \sigma^- c$ where $\mu^- = fN_{\text{neuron}}J^-/2$ and $\sigma^- = J^-\sqrt{fN_{\text{neuron}}/4}$. The total input is therefore approximately Gaussian distributed with mean and standard deviation given by

$$\mu = \frac{fN_{\text{neuron}}}{2}(J^+ + J^-), \tag{S.60}$$

$$\sigma = (J^+ - J^-)\frac{\sqrt{fN_{\text{neuron}}}}{2}. \tag{S.61}$$

A potentiated event will only occur in the downstream stage if $\theta = \theta_h$ and $h_i > \theta_h$ and a depressing event will only occur if $\theta = \theta_l$ and $h_i < \theta_l$. The probability that the fN synapses associated with the postsynaptic activity of a single neuron are changed is therefore

$$\phi = \frac{1}{2}\left(1 - \frac{1}{\sqrt{\pi}}\int_{\frac{\theta_l-\mu}{\sqrt{2}\sigma}}^{\frac{\theta_h-\mu}{\sqrt{2}\sigma}} dze^{-z^2}\right), \tag{S.62}$$

which, if the low and high thresholds are equally spaced from the mean can be written

$$\phi = \frac{1}{2}\left(1 - \text{erf}\Big(\frac{\theta-\mu}{\sqrt{2}\sigma}\Big)\right). \tag{S.63}$$

From the analysis of the previous section it is clear that the probability of a synapse in stage k being updated during the transfer process is not simply equal to the intrinsic learning rate q_k. Rather, for a transfer process of T replays, it is equal to

$$\bar{q}_k = (1 - e^{-q_k\phi fT}), \tag{S.64}$$

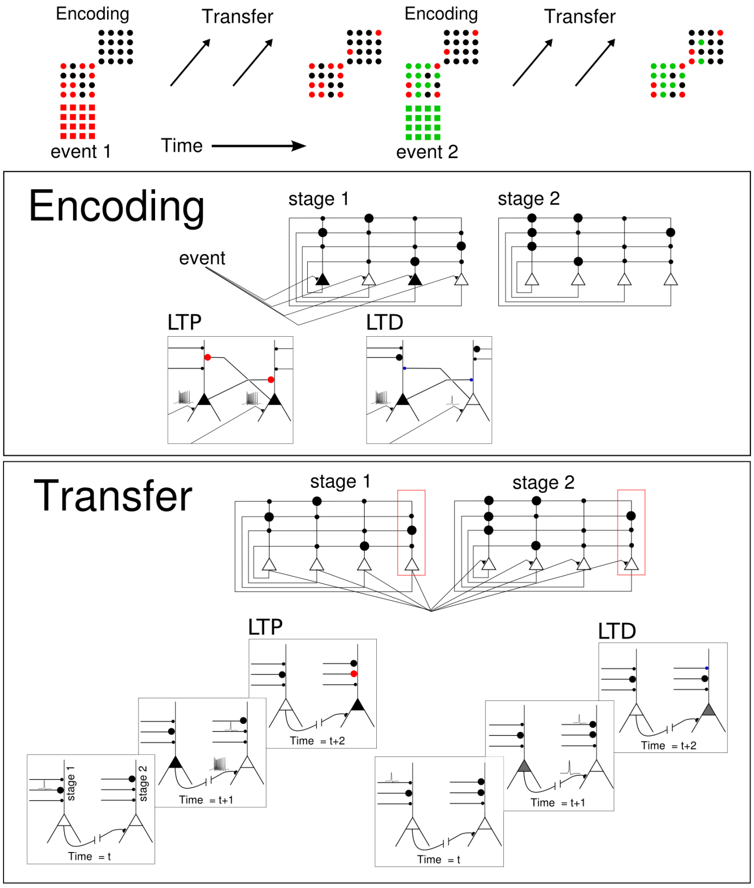

Fig. S.16: The neuronal model of memory consolidation. Top: Plasticity events, in this case patterns of neuronal activation, cause memories to be encoded through a Hebbian plasticity rule. Before the next memory is encoded a transfer process copies patterns of synaptic weights from one stage of the neuronal memory system to the next, downstream stage. Middle: A pattern of neuronal activation is imposed during encoding. A Hebbian learning rule leads to potentiation (depression) in the synapses connecting neurons with the same (different) activity. Bottom: An illustration of the transfer process for $f = 1/N_{\text{neuron}}$. LTP occurs whenever a presynaptic activation leads to postsynaptic firing. Otherwise LTD occurs.

which, for $q_k \phi f T \ll 1$ can be written

$$\bar{q}_k = q_k \phi f T. \tag{S.65}$$

Eq.S.64 is compared to numerical simulation of the full neuronal model for various values of f in Fig.S.17.

5.1.4 The fraction of synapses correctly transferred during replay

Of the total number of synapses transferred during replay, some fraction will be errors. The fraction of correctly potentiated synapses is just the fraction of the area under the curve greater than θ_h which is due to synapses in the potentiated state. It is also the ratio of the expected number of potentiated synapses to the total number of activated synapses per neuron, i.e. $\psi = E(k^+)/(fN)$. The expected number of potentiated synapses is just

$$E(k^+) = \frac{\int_{k^*}^{\infty} dk \cdot k e^{-\frac{(k-\mu)^2}{2\sigma^2}}}{\int_{k^*}^{\infty} dk e^{-\frac{(k-\mu)^2}{2\sigma^2}}}, \tag{S.66}$$

where $k^* = \frac{\theta_h - fNJ^-}{J^+ - J^-}$ is the minimal number of potentiated synapses needed to exceed the high threshold θ_h. Finally, we find that

$$\psi = \frac{1}{2} + \frac{1}{\sqrt{2\pi f N_{\text{neuron}}}} \frac{e^{-\xi^2}}{\text{erfc}(\xi)}. \tag{S.67}$$

This formula holds also for depressed synapses if the thresholds are equally spaced from the mean input. Eq.S.67 reaches a value of one when $\theta = fNJ^+$ which is the maximum possible input. Eq.S.67 is shown in Fig.S.18 for various values of f.

The effective learning rate during transfer

From the previous sections it is clear that the effective learning rate depends on the intrinsic learning rate times the number of replays, times a constant which depends on the details of the transfer process. The probability of correctly updating a synapse is then $\psi \bar{q}_k$ and an incorrect update occurs with a rate $(1-\psi)\bar{q}_k$.

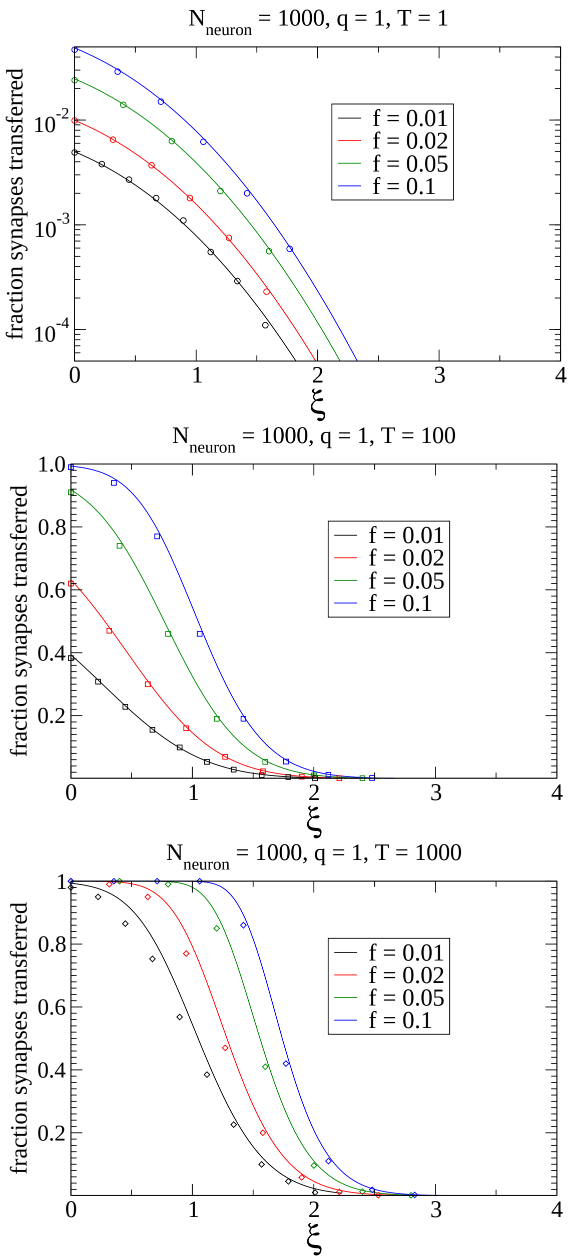

Fig. S.17: The fraction of synapses transferred, or transfer rate $\bar{q}$ as a function of $\xi = (\theta - \mu)/(\sqrt{2}\sigma)$ for $N_{\text{neuron}} = 1000$ neurons in the downstream stage and $q = 1$. Here $f = 0.01$ (black), 0.02 (red), 0.05 (green) and 0.10 (blue). The transfer rate is plotted for $T = 1$ (top), $T = 100$ (middle) and $T = 1000$ (bottom, note log scale on y-axis). $J^+ = 5$ and $J^- = 1$. Symbols are the average of 100 realizations of the full neuronal model. Lines are from Eq.S.63.

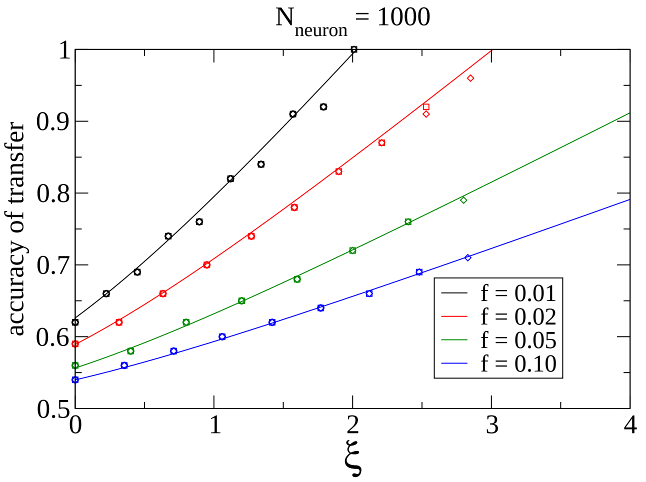

Fig. S.18: The fraction of correctly transferred synapses ψ. The symbols and all parameters are from the same simulations as in Fig.S.17. Note that ψ is not a function of T and therefore the symbols for each value of f collapse onto the same curve for different Ts. Lines are from Eq.S.67.

5.2 Meanfield model

The previous synaptic meanfield model still holds with one important correction. We must now take errors into account. In addition, the learning rates for stages $k > 1$ are now taken to be the effective learning rates calculated from the previous section. The probability of a synapse in stage 2 being in the potentiated state at time $t + 1$ is

$$p_2^+(t+1) = p_2^+(t)(1-p_d) + p_2^-(t)p_p, \tag{S.68}$$
$$p_d = \bar{q}_2 p_1^-(t)\psi + \bar{q}_2 p_1^+(t)(1-\psi), \tag{S.69}$$
$$p_p = \bar{q}_2 p_1^+(t)\psi + \bar{q}_2 p_1^-(t)(1-\psi), \tag{S.70}$$

where p_d and p_p are the probabilities of a depressing or a potentiating event respectively. This simplifies to

$$\dot{p}_2 = \bar{q}_2\psi(p_1 - p_2) - \bar{q}_2(1-\psi)(p_1 + p_2), \tag{S.71}$$

where we have taken the continuous time limit, dropped the + and subtracted off the equilibrium value of 1/2. The meanfield equation for the first stage is unchanged from the synaptic case as are the initial conditions. From

Eq.S.71 it is clear that the neuronal model approaches the performance of the synaptic model as $\psi \to 1$. However, in this limit we also have $\phi \to 0$, i.e. the transfer rate goes to zero. Therefore, to achieve good performance, ψ should be close to one, but this requires increasing T to compensate for low ϕ. That is, accurate transfer takes a large number of replays.

5.3 Build-up of correlations for many stages

Numerical simulations show that the model, as is, tends to generate strong correlations in the synaptic weights of synapses impinging on the same neuron. Specifically, at sufficiently long times each column in the synaptic matrix tends towards either an all-potentiated state or an all-depressed state. This build-up of correlations is more pronounced for more downstream stages for reasons which will be described below.

During replay, a fraction f of neurons is activated, this produces a subthreshold input in postsynaptic cells which is approximately Gaussian with mean and variance

$$\mu = \frac{fN_{\text{neuron}}}{2}(J^+ + J^-), \tag{S.72}$$

$$\sigma^2 = \frac{fN_{\text{neuron}}}{4}(J^+ - J^-)^2. \tag{S.73}$$

This calculation assumes that the probability of a synapse being in the potentiated or depressed state is simply 1/2. This is true on average, but if we consider one particular dendritic tree, i.e. the synapses which contact a particular neuron, there will be some deviation from this. That is, there will not be *exactly* $N/2$ potentiated and $N/2$ depressed synapses (ignoring autapses). So, this means that when a fraction f of neurons is stimulated, the true input to a cell will have mean and variance

$$\mu = fN_{\text{neuron}}((\frac{1}{2} + \epsilon)J^+ + (\frac{1}{2} - \epsilon)J^-), \tag{S.74}$$

$$\sigma^2 = fN_{\text{neuron}}(\frac{1}{2} + \epsilon)(\frac{1}{2} - \epsilon)(J^+ - J^-)^2, \tag{S.75}$$

which can be written

$$\mu = \frac{fN_{\text{neuron}}}{2}(J^+ + J^-) + \epsilon\frac{fN_{\text{neuron}}}{2}(J^+ - J^-), \tag{S.76}$$

$$\sigma^2 = \frac{fN_{\text{neuron}}}{4}(J^+ - J^-)^2(1 - 4\epsilon^2). \tag{S.77}$$

where $\epsilon = \frac{\delta}{N_{\text{neuron}}}$ and δ is the excess number of potentiated pre-synapses impinging on one neuron beyond $N_{\text{neuron}}/2$ so $\epsilon \in \{-1/2, 1/2\}$. Of course, we expect ϵ to be small. In fact, since the synapses are updated essentially as a Poisson process with the probability of potentiation and depression both being 1/2, the expected number of potentiated synapses is $N_{\text{neuron}}/2$ and the standard deviation is $\sqrt{N_{\text{neuron}}}/2$ which means $\epsilon \sim \frac{1}{2\sqrt{N_{\text{neuron}}}}$. However, it doesn't matter how small ϵ is, it will always lead to large fluctuations in downstream synaptic states if: 1 - for a fixed number of stages there are sufficiently many replays or 2 - for a fixed number of replays there are sufficiently many stages. The reasons is that even a small excess of potentiated synapses leads to an imbalance in potentiation over depression in the next stage. This effect is accentuated by the fact that plasticity is completely driven by the tails of the input distribution. Therefore sufficiently many replays will always make the ϵ in the next stage bigger than in the current one, making the system unstable. This can be seen in Fig.S.19 where the input to each neuron is plotted, averaged over 5000 replays. The inputs to neurons in stages 2, 4 and 6 are shown. The inputs to stage 2 are clearly tightly peaked around 30, since here $f = 0.01$, $N_{\text{neuron}} = 1000$, $J^+ = 5$ and $J^- = 1$. The inputs to stages 4 and 6 show much greater variability with those for stage 6 clearly grouping around 10 and 50, indicating that for each neuron all inputs are either depressed or potentiated respectively. The mean averaged over the network is still 30.

A simple solution to avoid such a build-up of correlations is to allow for a variable threshold in the plasticity rule. Specifically, the threshold should compensate for changes in excitability as in the BCM rule. The most trivial implementation of this is to subtract off the value $\epsilon \frac{f N_{\text{neuron}}}{2}(J^+ - J^-)$ from the input to any cell. This is equivalent to shifting both thresholds by the same amount. This does not take into account the effect of correlations on the width of the input distribution, but that effect is order ϵ^2 and is negligible. Fig.S.20 shows the same simulation as in Fig.S.19 but with the corrected input. The fluctuations are clearly stabilized in this case.

5.4 Neuronal readout of memories

We have described how the *memory transfer model* can be implemented in a simple network of McCulloch-Pitts neurons with two modes of activity: encoding and transfer. In this neuronal model, memories are still defined as the patterns of synaptic connectivity. These patterns are encoded by

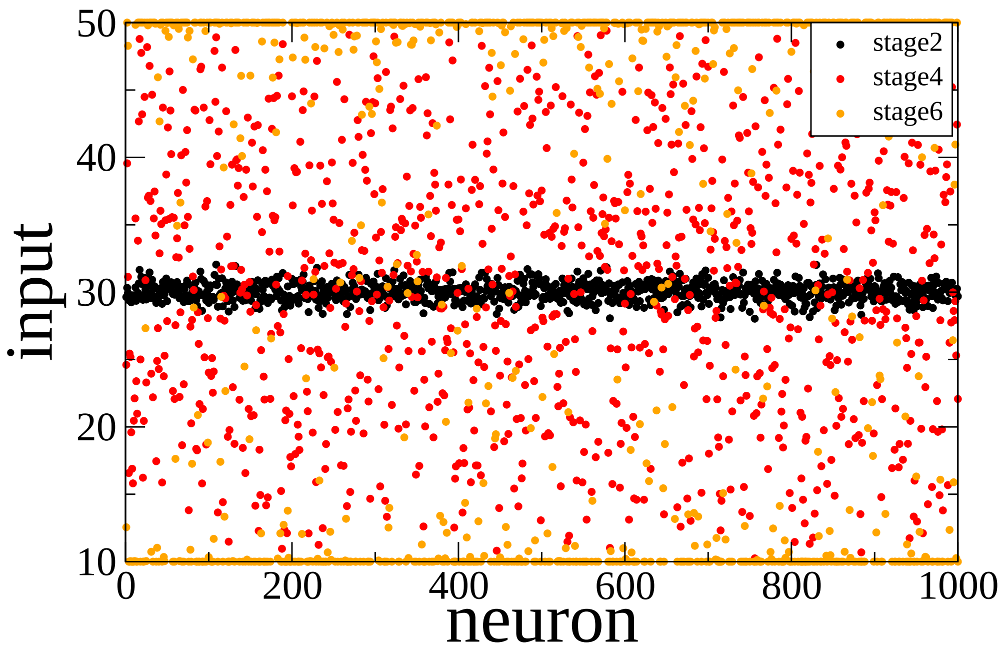

Fig. S.19: Inputs to cells becomes progressively split between purely depressed and purely potentiated.

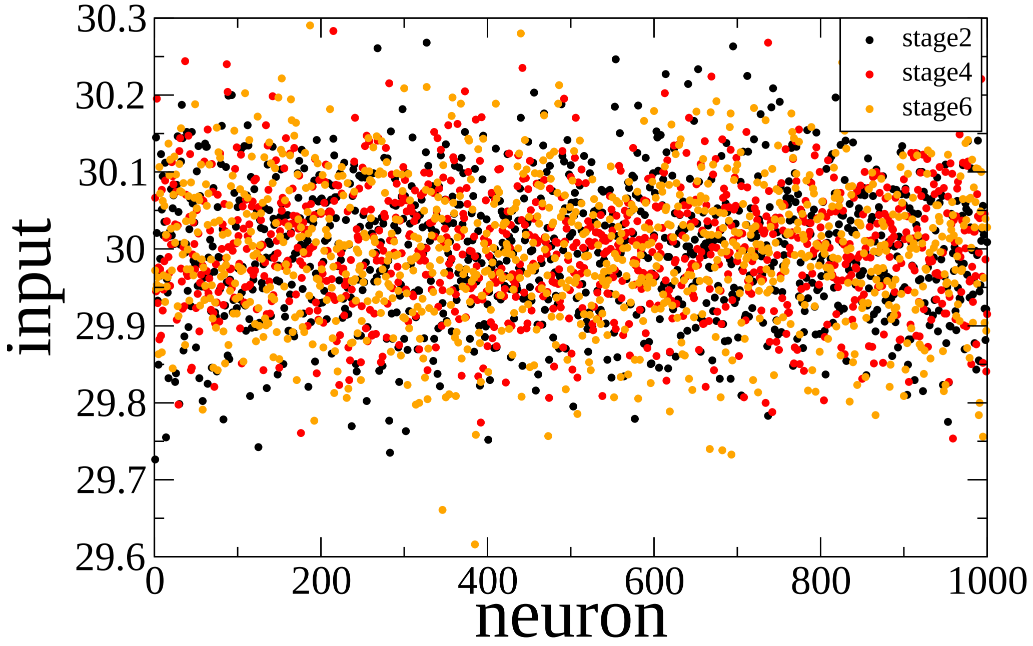

Fig. S.20: Correcting for the effect of fluctuations on the mean input stabilizes the system.

imposing a particular pattern of neuronal activity in the first stage during the encoding phase. Therefore we can associate a pattern of neuronal activity with every pattern of synaptic weights (memory). This allows us to consider a simple means of 'reading out' memories, by which we mean detecting a pattern of neuronal activation which has been previously used during the encoding phase. For this particular type of readout we drive one half of the neurons in stage k. The pattern of activation is random and may be a novel (not learned) pattern or may coincide with a previously learned pattern. Our task is to design a read-out circuit which can distinguish between these two types of patterns: novel or learned. Therefore this is a recognition task.

Let us designate by the vector $\mathbf{s}^k(t)$ the state of neurons in stage k at time t, where for neuron i $s_i^k(t) \in \{0, 1\}$ and 1 means active. The activity of neuron i at a time $t+1$ depends on the recurrent synaptic connectivity. Specifically, the input current to neuron i is $h_i^k(t) = \sum_j J_{ij}^k(t) s_j^k(t)$, and if $h_i^k(t) > \theta$ then $s_i^k(t+1) = 1$. The question is, 'How does $h_i^k(t+1)$ depend on whether or not $\mathbf{s}^k(t)$ is a novel or a learned pattern?' If there is a systematic difference in this input current as a function of the novelty of the pattern of activation, then it is possible to recognize previously learned patterns.

As an illustrative case consider a stage with learning rate $q = 1$, i.e. every synapse is overwritten at every time step. Then

$$J_{ij}(t) = \frac{J^+ + J^-}{2} + \frac{J^+ - J^-}{2}\big(2s_i(t-1) - 1\big)\big(2s_j(t-1) - 1\big), \qquad \text{(S.78)}$$

where we have dropped the superscript k. Now we find that

$$h_i(t) = \frac{J^+ + J^-}{2}\sum_j s_j(t) + \frac{J^+ - J^-}{2}\big(2s_i(t-1) - 1\big)\sum_j \big(2s_j(t-1) - 1\big)s_j(t). \qquad \text{(S.79)}$$

The expected value of the input is

$$E(h_i) = \frac{N_{\text{neuron}}}{4}(J^+ + J^-) + (J^+ - J^-)(2s_i(t-1) - 1)\Big(E(\sum_j s_j(t-1)s_j(t)) - \frac{N_{\text{neuron}}}{4}\Big), \qquad \text{(S.80)}$$

and so clearly depends on the correlation between the patterns of activation at times $t-1$ and t. If they are perfectly correlated (anti-correlated) patterns, then $E_C(h) = \frac{N_{\text{neuron}}}{2}J^+$ if $s_i(t-1) = 1$ ($s_i(t-1) = 0$)and $E_C = \frac{N_{\text{neuron}}}{2}J^-$ if $s_i(t-1) = 0$ ($s_i(t-1) = 1$). If they are uncorrelated, then $E_U = \frac{N_{\text{neuron}}}{4}(J^+ + J^-)$. Also, in this simple example we also have that

$Var_C(h_i) = 0$ and $Var_U(h_i) = \frac{3}{8}(J^+ - J^-)^2 N_{\text{neuron}}$. Therefore in this simple case the distribution of inputs given novel patterns of activation is an approximate Gaussian with the mean and variance given above, while for a learned memory it is two delta functions centered to the left and right of the mean of the Gaussian. Therefore, the threshold for the McCulloch-Pitts neurons can be placed between the mean of the Gaussian and the rightmost delta function $E_U < \theta < E_C$. This will lead to many more neurons being active at time $t + 1$ whenever the pattern of activation is learned and not novel. A simple readout would then be to recognize a learned pattern if the sum $\sigma = \sum_i s_i(t + 1)$ is greater than a certain threshold.

In the general case ($q \neq 1$) the separation between the input distributions given a learned or a novel pattern of activation is not as large. In fact, after a pattern is learned (memory encoded), the difference $E_C - E_U$ will decrease exponentially in time with a time constant proportional to $1/q_k$ where q_k is the learning rate for stage k. Furthermore, $Var_C > 0$. Therefore, the task of recognition is a signal-detection problem given Gaussian distributions. In any case, it is clear that given any θ and any threshold for recognition of a learned pattern, some errors will be made. The probability of correct recognition will clearly decrease as a function of the age or SNR of the memory being tracked. This is illustrated in Fig.S.21 for the neuronal *memory transfer model* given the same parameter values as in Fig.6 of the main text. In Fig.S.21, the upper panel shows the SNR of each of the first five stages as well as the total SNR (black). The lower panel shows a recognition index x_{recog} using each of the individual stages as well as all of them combined (black). To make these curves, after each encoding/transfer process (one time step), each stage is probed with both a novel pattern of activation and the pattern which was learned at $t = 0$. Both of these patterns produce a response (here $\theta = E_U + StDev_U$). In this case we simply compare the responses and say that the larger response wins (that pattern is recognized). We then repeat the entire simulation 1000 times. The recognition index is the number of times the learned pattern led to a larger response minus the number of times the novel pattern lead to a larger response divided by 1000. Therefore $x_{\text{recog}} \in \{-1, 1\}$ and $x_{\text{recog}} = 0$ is chance level. No effort was made to optimize the readout in any way. Clearly, no errors occur when the SNR is large, and then the rate of errors increases with decreasing SNR.

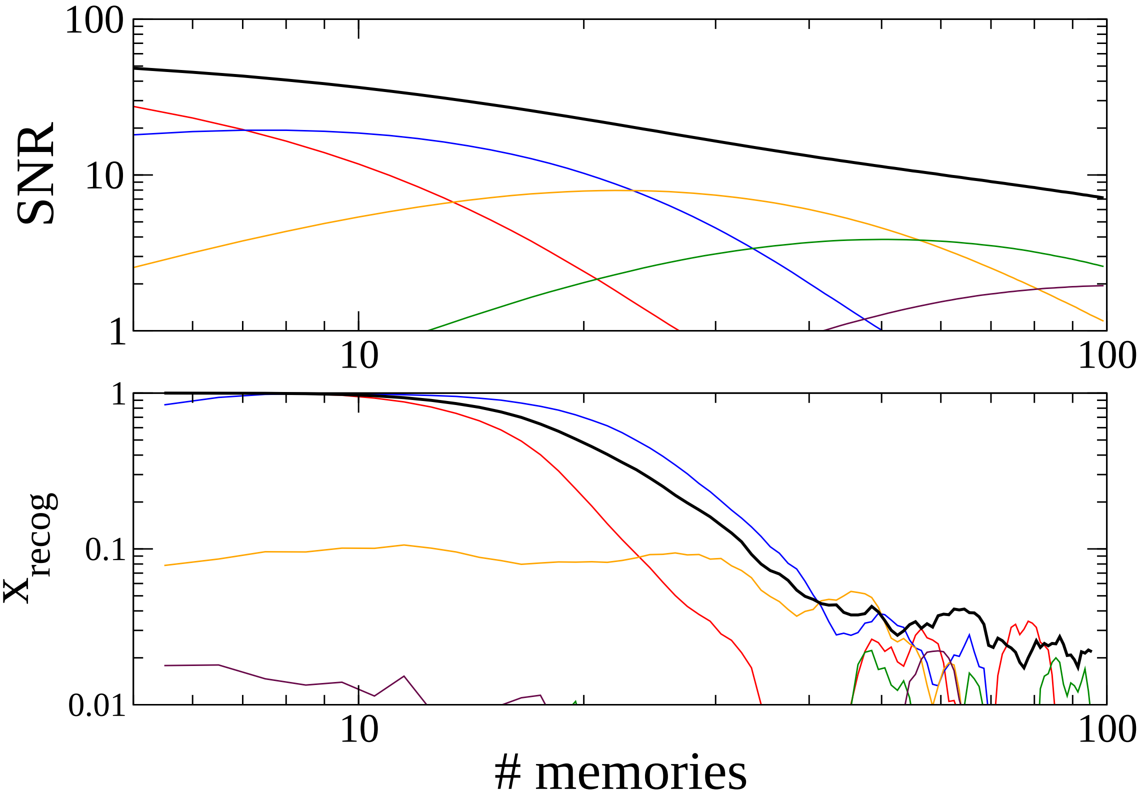

Fig. S.21: The SNR (top) and recognition index (bottom) for the neuronal memory transfer model. All parameters are the same as in Fig.6 of the main text. Curves are from all stages (black), stage 1 (red), stage 2 (blue) stage 3 (orange), stage 4 (green) etc.

6 A neuronal memory transfer model with random projections

Here we describe a modified neuronal memory transfer model in which we relax the assumption of one-to-one feedforward connection between stages. Our results are entirely numerical.

Once again there are n stages with N_{neuron} neurons in each stage. The new twist is that at each stage > 1 each neuron i receives input from a random subset of neurons in the previous stage. The probability of forming such a feedforward connection is p_{ff} so each neuron receives on average $p_{ff}N_{\mathrm{neuron}}$ connections. Recurrent synapses are binary with values $J \in \{J^-, J^+\}$ while feedforward synapses are all taken identical with $J = J_{ff}$. Recurrent connections are sparse with a probability of connection of p_r. The simulations are carried out in the following steps:

6.1 Initializing matrices

We first initialize all recurrent matrices by randomly assigning synaptic weights. For the connection from cell j to cell i, J_{ij} is non-zero with probability p_r and zero otherwise. If it is nonzero then it is assigned a value J^+ with probability $1/2$ and is set to J^- otherwise. Feedforward matrices are initialized similarly. For the connection from cell j in stage k to cell i in stage $k+1$ I set $J = J_{ff}$ with a probability p_{ff} and to zero otherwise.

We encode a memory by randomly stimulating a subgroup of neurons in stage 1. The state of a neuron i in stage 1 at a time t is given by $s_i^1(t) \in \{0, 1\}$. Thus at time $t = 0$ we set $s_i^1(0) = 1$ with a probability f_m (m for memory). We activate another randomly selected subgroup of neurons at time $t = 1$, again with probability f_m. For all simulations $f_m = 1/2$. If neuron j at time $t = 0$ and neuron i at time $t = 1$ are in the same state we set $J_{ij} = J^+$ with a probability q_1. If the activities are different then we set $J_{ij} = J^-$ with a probability q_1. If there is no connection between the cells (due to sparse connectivity) we do nothing. At the same time we initialize the template of the memory in stage 1. This is the same as the preceding procedure except that the 'effective' $q_1 = 1$.

Initializing the templates for the downstream stages is more involved. When we stimulate the randomly chosen subset of neurons in stage 1 at time $t = 0$, there is some subthreshold input to cells in stage 2 due to the

feedforward connectivity. This input is approximately Gaussian with mean $\mu_m = f_m p_{ff} J_{ff} N_{\mathrm{neuron}}$ and variance $\sigma_m^2 = J_{ff}^2 f_m p_{ff}(1 - p_{ff}) N_{\mathrm{neuron}}$. To maintain the same level of coding, i.e. ensure that only about half of the neurons in stage 2 are active, we set a threshold $\theta_m = \mu_m$ such that for a neuron i in stage 2 receiving an input h_i, if $h_i > \theta_m$ we set $s_i^2(0) = 1$, otherwise it is set to zero. The same holds true at time $t = 1$. Once we have done this procedure for times 0 and 1, we can set the weights for the template. If $s_j^2(0)$ and $s_i^2(1)$ have the same state, then we set $J_{ij} = J^+$ with probability 1 (unless there is no connection) and if the states are different we set $J_{ij} = J^-$.

6.2 Calculating overlap

This is just a procedure which can be carried out at anytime. If the recurrent connection from cell j to cell i in stage k is R_{ij}^k and the template 'connection' between the same cells is T_{ij}^k then the overlap $m^k = \frac{c}{p_r N_{\mathrm{neuron}}^2}$ where c is the number of nonzero elements which are the same. The strength of the memory in stage k is then $2m^k - 1$. This removes the overlap due to chance (1/2). Then the $SNR_k = \sqrt{N}(2m^k - 1)$ where N is the number of synapses in one stage. This is the quantity which is plotted in Fig.S.22.

6.3 Replay

For each replay, we activate a random subset of neurons. For the sake of argument we will discuss replay from stage 1 to stage 2. For a replay at time t neuron j in stage 1 is set to 1 with a probability f. This results in fN neurons being active on average. This activation causes some postsynaptic input within stage 1 due to the recurrent connections. This input is approximately Gaussian with mean and variance given by

$$\mu_r = f p_r N_{\mathrm{neuron}} \frac{(J^+ + J^-)}{2}, \tag{S.81}$$

$$\sigma_r^2 = f p_r \frac{N_{\mathrm{neuron}}}{4} \left((J^+ - J^-)^2 + (1 - p_r)(J^+ + J^-)^2 \right), \tag{S.82}$$

where the first term of the variance is due to differences in the synaptic weights and the second term is due to quenched randomness due to sparse connectivity. Obviously the input to neuron i, h_i will be highest if there is a

significant overlap between the random pattern of activity and the synapses potentiated by a previous memory. It will be least when the random pattern is anti-correlated with the potentiated synapses, i.e. it is correlated with the synapses depressed by a previous memory. So as before we set two thresholds, one high and one low, θ_h and θ_l. We choose one of these two thresholds randomly with probability 1/2 at the beginning of the replay. In either case, if $h_i > \theta$ then $s_i^1(t+1) = 1$ and otherwise it is zero. The fraction of neurons active at time $t+1$ can be determined from the threshold and the Gaussian input distribution defined by Eqs.S.81 and S.82. It is clear that when there is a high threshold very few neurons will be active, and when there is a low threshold very many neurons will be active.

The activity in stage 1 at times t and $t+1$ lead to subthreshold input to neurons in stage 2 due to the feedforward connections. Again this input will be approximately Gaussian. The input distribution at time t has mean and variance

$$\mu_{ff}(t) = f p_{ff} J_{ff} N_{\text{neuron}}, \tag{S.83}$$
$$\sigma_{ff}^2(t) = J_{ff}^2 f p_{ff}(1-p_{ff}) N_{\text{neuron}}, \tag{S.84}$$

whereas the input distribution at time $t+1$ has mean and variance

$$\mu_{ff}(t+1) = \frac{\mu_{ff}(t)}{fN}\sum_i s_i^1(t+1), \tag{S.85}$$
$$\sigma_{ff}^2(t+1) = \frac{\sigma_{ff}^2(t)}{fN}\sum_i s_i^1(t+1), \tag{S.86}$$

where the number of active neurons in stage 1 at time $t+1$ depends on the details of the recurrent dynamics as discussed above.

Now, the idea is to set thresholds for the feedforward connections so as to correctly transfer some of the synapses. First we will assume that we have chosen θ_h for the recurrent dynamics. The handful of neurons which become active in stage 1 at time $t+1$ are those which overlap maximally with the random pattern presented at time t. Thus we want to select the neurons in stage 2 that receive their input predominantly from these two subsets of neurons. This should give the maximal overlap in the transformed space of stage 2 as well. We do this by setting high thresholds. However, we cannot set the threshold to be the same for both time steps since the input statistics are completely different. What we do is set the threshold to be

$\theta_{ff}(t) = \mu_{ff}(t) + \alpha\sigma_{ff}(t)$ where α can be varied to get different transfer and error rates. The means and variances are just from Eqs. S.83-S.86. Now we potentiate the connection from cell j to cell i in stage 2 with probability q_2 if $s_j^2(t) = 1$ and $s_i^2(t+1) = 1$. If the recurrent threshold is θ_l then we still want the neurons in stage 2 at time t to receive their input predominantly from the neurons in stage 1 constituting the random pattern. However, now it is the neurons in stage 1 which *did not* fire at time $t + 1$ which are maximally correlated with the depressed part of the memory. Therefore, I want to isolate the neurons in stage 2 at time $t+1$ which predominantly receive inputs from those neurons. We do this by setting a low threshold. Those neurons in stage 2 which despite the low threshold still do not fire must be receiving a significant share of connections from the neurons which are not active in stage 1. Now we depress the connection from cell j to cell i in stage 2 with probability q_2 if $s_j^2(t) = 1$ and $s_i^2(t + 1) = 0$.

We repeat the replay process T times. We then encode a new memory in a way analogous to the first. Figure S.22 shows the results of a simulation with two stages, each with 1000 neurons and random projections with feedforward sparseness of 0.1. The top shows the fraction of synapses transferred per replay which are actually updated correctly. The fluctuations are large but this process does better than chance on average. The resulting memory traces are shown in the bottom panel.

References

[1] S. Fusi, P. J. Drew, and L. F. Abbott. Cascade models of synaptically stored memories. *Neuron*, 45:599–611, 2005.

[2] Stefano Fusi and L. F. Abbott. Limits on the memory storage capacity of bounded synapses. *Nat Neurosci*, 10(4):485–493, Apr 2007.

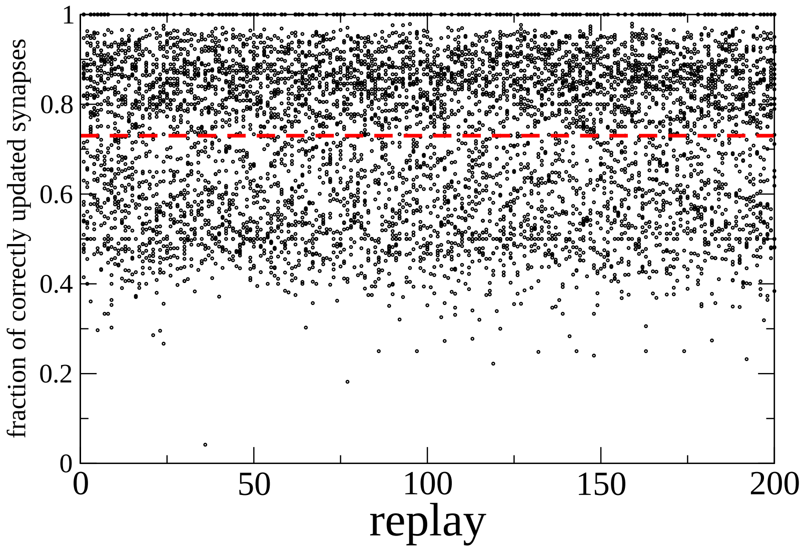

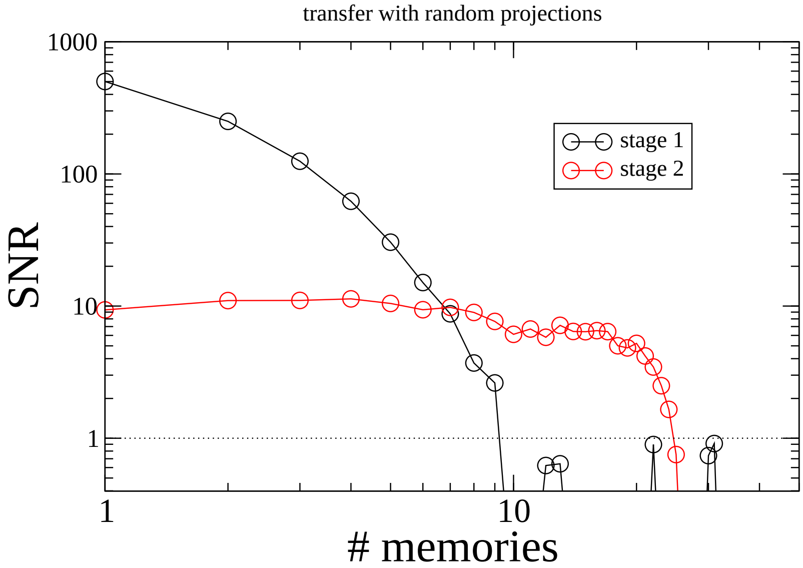

Fig. S.22: Upper figure: The fraction of updated synapses in stage 2 which were updated to the correct state (according to the template) for each replay over 200 replays per memory and 30 memories in this case. The dashed red line shows the mean which is at 0.73. Chance is 0.5. Bottom figure: The overlap between the synaptic matrix in stages 1 and 2 and their respective templates in a system with random projections. Parameter values are: n=2, N=1000, $p_r = 1$, $J^+ = 5$, $J^- = 1$, $J_{ff} = 2$, $p_{ff} = 0.1$, $f_m = 0.5$, $f = 0.5$, $\theta_h = \mu_r + 2\sigma_r$, $\theta_l = \mu_r - 2\sigma_r$, $\alpha = 2$ (see description of feedforward thresholds in text), $q_1 = q_2 = 0.5$, $T = 200$. Note that the overlap in stage 2 is zero for $t = 0$.
